# Weapon detection with FMR-CNN and YOLOv8 for enhanced crime prevention and security

**DOI:** 10.1038/s41598-025-07782-0

**Published:** 2025-07-23

**Authors:** Shanthi P, Manjula V

**Affiliations:** School of Computer Science and Engineering, Vellore Institute of Technology, Chennai, 600 127 India

**Keywords:** Closed-circuit television (CCTV), Computer vision, Deep learning, Faster region convolutional neural network (Faster-RCNN), Mask region convolutional neural network (Mask-RCNN), You only look once (YOLO), MobileNetv3, Engineering, Mathematics and computing

## Abstract

In modern days, increasing weapon-related threats in public places have created an immediate need for intelligent surveillance systems to detect crime in real-time. Traditional surveillance systems have struggles with recognizing small objects, occlusion, and the time it takes to respond, which makes them ineffective in crowded and fast-changing situations. To overcome these challenges, the suggested system combines closed-circuit television (CCTV) surveillance cameras with advanced deep learning methods, image processing, and computer vision techniques for real-time crime prediction and prevention. This study proposes a hybrid deep learning framework that merges a Faster region convolutional neural network and Mask Region Convolutional Neural Network, named FMR-CNN. The novel approach FMR-CNN represents a significant advancement towards improving object recognition and segmentation of images and videos. It has been combined with YOLOv8 to increase the real-time detection speed and localization accuracy significantly. Such a combination enables the concurrent utilization of high-resolution spatial context information and rapid frame-wise predictions, thus making it well-suited for continuous video surveillance tasks. The model was trained and tested on a five labeled class annotated dataset, where MobileNetV3 features are extracted to simulate real-world surveillance conditions. Experimental results show the hybrid model attains detection accuracy of 98.7%, average precision (AP) of 90.1, and speed of 9.2 frames per second (FPS), and generalizes to varied lighting, occlusion, object scales, and reduced computational complexity, making it highly effective for crime prevention. Using these models benefits police departments and law enforcement agencies, as it allows them to detect criminal offenses earlier and avoid untoward situations.

## Introduction

Advanced deep learning models integrated with FMR-CNN and YOLOv8 are used for real-time threat detection in a CCTV setup and very accurately identify such threats very fast and prevent a crime automatically. This work, therefore, presents a fresh approach by introducing a kind of AI that dramatically improves the responsibilities of improving the prediction and prevention of crime benchmarks better than the manual benchmarks of surveillance systems. Distinctly from largely single detection models present in the literature, the research at hand increases the detection accuracy and minimizes false positives and computational load by integrating multiple deep learning frameworks, such as FMR-CNN and YOLOv8. CCTV, with its many surveillance capabilities, plays a crucial role in the prediction of crime activities: inhibition, monitoring, collection of evidence, identification, prevention, post-event analysis, public safety, security, and road safety responses^1^. It stands out as useful for seamless observation of vital information and aiding in various areas to enhance security in order to protect the environment and property. Several limitations face CCTV surveillance systems, such as human or operator fatigue, subjective bias, and delayed threat responses. Therefore, in consideration of the stated limitations, this particular study presents an AI-enabled strategy to detect weapons in real time and with high accuracy and does away with human judgment in security operations.

Military settings initially used CCTV for observation and surveillance, developing systems with analog cameras and monitors connected by coaxial cables. Currently, CCTV is integrated with AI and analytics. Artificial intelligence aspects such as facial recognition, object detection, person re-identification, and characteristic analysis permit automated monitoring, live alerts, and advanced search functionalities. This integration enhances situational awareness and security personnel. The use of camera networks in public places such as malls, roadways, railways, airways, schools, and colleges through the interpretation of movements proceeds further in surveillance techniques and increases the demand for public safety. Although camera networks acknowledge large amounts of regular video image information, they are useful for multitudinous camera tracing along with judiciary inspection.

Humans easily get tired during continuous observation, and different people may think that different things are not the same, resulting in different biases. Tying together the weapon-detection system with the CCTV camera is very useful in identifying the problem. There are two approaches for weapons detection with images and videos captured from CCTV: body-proportional square classification and object detection in a region-proposal network (RPN). Many techniques have been proposed, including YOLOv3, YOLOv4, Faster Region-Based CNN Inception-ResNetV2 (FR IRv2)^2^, and single-shot detector MobileNetV1^3^. Object detection systems using deep learning techniques execute these tasks in three stages: identification, recognition, and separation from the video stream^4^. To achieve and distinguish between weapon and no-weapon activities in CCTV surveillance, YOLOv5 is combined with a convolutional neural network (CNN)^5^. In particular, deep learning systems (DLs) have been used to determine the dominant role of video-based Person Re-Identification (Re-ID) techniques^6,7^.

According to an evaluation, the annual firearm-related death rate in India in 2019 was 3.22 per 100,000 people, with 90% of these incidents involving illegal firearms^8^. Crime surveillance and detection are the most important aspects of public safety. An ancestral CCTV surveillance system works on human observation, consuming much more time, being prone to human mistakes, and having less prediction of real-time crime activities. To overcome these shortcomings, this research suggests an innovative weapon detection system that combines FMR-CNN and YOLOv8, improving the accuracy, speed, and effectiveness of crime prediction and prevention significantly. The proposed system enhances the detection and classification of objects with the use of models such as Faster R-CNN refinement of object proposal regions, Mask R-CNN for precise segmentation, and YOLOv8 for fast detection, which provides a highly responsive surveillance system. Initially, the weapon detection system starts with data preprocessing, augmentation by resizing, normalization, and transformation through rotation, flipping, and contrast adjustment in images and video frames. After completing preprocessing, the system goes with feature extraction and region proposal involved in Faster R-CNN, which uses the backbone of CNN to extract feature maps from images. It utilizes a Region Proposal Network (RPN) to identify potential Regions of Interest (RoIs) with weapons inside the RoI. These ROIs undergo ROI pooling, which standardizes their size so that classification and bounding box regression can happen; detected weapons with bounding boxes ensure the accuracy of object classification. The ROIs from Faster RCNN are further refined using the model of Mask RCNN; it generates pixel-wise segmentation masks, which allow the system to precisely define the shape and contours of weapons. This segmentation method reduces false positives by distinguishing weapons from visually close objects in littered backgrounds. YOLOv8 is the backbone of the real-time detection algorithm, processing the full frame once, after which bounding boxes and class labels are immediately predicted. Non-maximum suppression eliminates low-confidence detections in order to maintain efficiency and ensure a fast and robust surveillance performance. In training and optimization, YOLOv8 is trained with the help of high-resolution datasets through a technique called transfer learning in order to improve performance in real-world applications, e.g., through CCTV. FMR-CNN utilizes a pre-trained CNN backbone, such as MobileNetv3, in order to perform the feature extraction. Cross-entropy loss is used in order to optimize classification, while smooth L1 loss is utilized with the bounding box regression to increase the precision.

At the end of the training phase, the system is launched to enable real-time monitoring of criminal activity and to improve predictive analytic capabilities. Video analysis enables continuous detection and tracking on a frame-by-frame basis for possible weapons. It employs MobileNetV2 for behavior assessment, leading to the detection of suspicious activities and the prediction of crime. The threat assessment system does a compilation of the different detections across consecutive frames in order to develop a complete evaluation, which also allows for a reduction in false alarms and increased effectiveness of the response strategies. The system also has an automated alarm system that guarantees timely response. The moment a weapon is detected, alarms are triggered instantly, together with corresponding images and video clips. The alarm is transmitted through an API to law enforcement agencies, allowing swift response. Confidence scores are employed in the determination of the severity of detection to guarantee that confirmed threats only trigger a response, thus preventing unnecessary interventions. The contributions of this paper are structured as follows:

- Weapon Detection System: This work improves the reliability of CCTV surveillance systems by integrating deep learning methods due to the delivery of high accuracy in real-time applications.
- High-Precision Object Segmentation and Classification: Utilizing FMR-CNN guarantees precise localization, segmentation, and classification of weapons, thereby reducing the likelihood of false positives.
- Real-Time Detection with YOLOv8: With YOLOv8, the system detects weapons effectively during live surveillance and feed monitoring sessions in real time.
- Automated Threat Notification System: This system automatically notifies law enforcement officers about the existence of a weapon, facilitating prompt action and discouraging criminal behavior.
- Enhanced Computational Efficiency: It enables real-time processing at a low computational expense, making it appropriate for use in diverse security settings.

Overall, the proposed weapon detection system significantly improves real-time surveillance and increases public safety by reducing the number of false alarms, supporting the investigative activities of law enforcement agencies, and offering flexibility in various security situations. The use of various deep learning models makes this system a very effective tool for modern AI-based security systems.

This paper is divided into various sections in an effort to give a systematic review of the proposed system. In Section review of related studies and techniques in weapon detection and surveillance through deep learning, highlighting areas of research gaps. Section of proposed technique, presents experimental analysis, discussion of results, and finally,concludes the study by presenting its contributions and proposing future enhancements.

## Related works

Various modern surveillance mechanisms utilize geocentric features to identify the background along with machine learning-aided deep learning technologies. Numerous recent scholarly works have explored areas ranging from weapon detection, facial recognition, anomalous behavior detection, and human interaction recognition panels. The elements of machine learning and deep learning approaches relevant to this discussion are surveyed in this part. For weapon detection, various already existing methods follow general principles in object detection, with styles based on different CNN architectures. The web is an extensive study, including a diverse set of techniques based on the principles of YOLO (versions 2, 3, 4, 5, 7, and 8) coupled with Mask R-CNN, Faster R-CNN, VGG16, VGG19, ResNet, and MobileNet, which are key in recognizing and classifying weaponry. Such algorithms, such as the Faster R-CNN and Mask R-CNN, are recognized for their accuracy. However, what sets YOLO apart is its stunning speed, which serves live detection. Arockia Abins et al.^9^ developed a immediate response weapon system by using the deep learning algorithm and achieved an active speed of 0.05 seconds per frame. This combat detection model utilizes CNNs and the PELSF-DCNN classifier for weapon detection with minimal false positives. A behavior analysis module is used to analyze the movement of the detected objects for judging possible threats, while the alerting module notifies the authorities in case of incidents. Sliding window processes and feature selection techniques increase the system performance.

Gyanendra Verma et al.^10^ performed automatic gun detection from crowded places via faster R-CNN, the deep convolution neural network DCCN, a state-of-the-art faster RCNN-based convolution neural network model, through transfer learning. Guanbo Wang et al.^11^ developed an improved YOLOv4 model for real-time CCTV automatically detecting weapons, which improved object detection in terms of security and counter-terrorism. Non-Uniform-You Only Look V4 backbone with spatial cross-stage partial ResNet (SCSP-ResNet), receptive field amplification module, and Fusion-PaNet (F-PaNet) module. The pruning model reduces the parameters and weight file size, increasing real-time performance without a significant loss of accuracy, and the utilization of the k-means clustering algorithm improves model accuracy and recall for small objects. Bushra S N et al.^12^ detected weapons at all common places, including sensors, to analyze anticipated activities. The YOLOv5 model appeals to all regions of the input image and determines the highest score for region detection, a time-consuming task, and misdemeanor activities are detected and fully clear vision in a crowd. A computer vision developer model called Roboflow helps in data collection, preprocessing, and training model techniques and particularly deploys custom datasets with easy flow. Goenka et al.^13^ used surveillance security cameras and the drawbacks of traditional object detection methods and the complexity of the environment and concession between speed and quality in high-resolution LiDAR imaging^14^. The authors proposed a deep learning-based automatic gun detection system using the Mask RCNN model with and without applying Gaussian de-blurring features, demonstrating increased precision, recall, accuracy, and F1 scores with de-noising features. The gun detection technique for an effective video surveillance system and proposed future directions for improving the model performance, such as combining various categories of guns in the dataset and exploring advanced models such as YOLOv4 for real-world applications, are proposed. An algorithm for object detection with the help of deep learning techniques on large datasets of images to increase the model’s ability to classify objects highly^15^. In a study by Khalid Sulaiman et al.^16^, a computer vision-based smart surveillance system using YOLOv6 for automated weapon detection was developed. It aims to address the global increase in weapons and violence by detecting visible weapons and sending real-time alerts to authorized persons or departments with the help of DL algorithms for object detection and object recognition.

Pavithra et al.^17^ proposed that makes use of object identification strategies, specifically YOLOv7, to identify targets and their respective trajectories in video data; with considerations to time gaps between movements and a categorization feature. The COCO datasets analyze distinct video scenarios, such as abnormal activity, burglary attempts, and false alarms. YOLO has performance measurements with a 93% precision, an F1 score of 94.42%, a recall of 94.71%, an accuracy of 95%. The YOLOv7-DarkVision architecture improves weapon detection in low-light situations and increases accuracy by integrating the YOLOv7 architecture. 92.5%^18^. Anthony Ortiz Ramon et al.^19^ intended to detect different types of weapons in public spaces to address the high rates of violence and insecurity in the region. By utilizing YOLOv3 and an efficient D0, YOLOv3 was proven to be the most effective in detecting firearms, with an accuracy of 0.80%. Hyper-realistic 3D models and internet searches with augmentation techniques to enhance security measures by accurately detecting weapons in city circumstances via advanced object detection techniques.

**Table 1:** Comparison of conventional approaches.

Author name and refs.	Methodology	Findings	Limitations
Tahir et al.^15^	YOLOv4	The work created a new weapon recognition system based on deep learning using YOLOv4 and CNN, whereby YOLOv4 demonstrates greater accuracy even if lengthier training sessions are involved. This study underlines the critical requirement of advanced surveillance to solve growing gun violence and suggests that automation could be crucial in saving lives and lowering the death rate.	While CNN struggles with accuracy despite more layers, revealing its shortcomings in great real-world data environments, the research shows a fine line between speed and accuracy in weapon detection systems; YOLOv4 improves Mean Average Precision (mAP) with larger datasets, while dataset size varies.
Shehzad Khalid et al.^16^	Faster RCNN and SSD	The work emphasizes the important part that feature extraction plays in anomaly and weapon detection. Several other frameworks for speeding up weapon detection include SSD and RCNN through bounding box generation and dataset training. It is important to mention that while SSD can do fast real-time detection, it gives in terms of accuracy.	This study reviews the very delicate balances between speed and accuracy in object detection models like Faster RCNN. Furthermore, real-time application of these models suffers some challenges, mainly due to the slow frame rate of Faster RCNN, which hampers its use wherever fast processing is of utmost importance.
Yadav Gupta.^20^	YOLOv7	Outstanding precision rates of 91.62% on the MAD dataset and 98.50% on the PDD dataset let the model surpass earlier studies. The emphasis will be on enhancing weapon detection technologies and creating real-time alert systems for useful application going ahead.	In high-resolution LiDAR imaging, complexity surrounds and concessions between speed and quality.
Pullakandam et al.^21^	YOLOv8	With a startling mean average precision (mAP) of 90.1%, the YOLOv8 model defeated YOLOv5 in weapon identification. YOLOv8 proved deep learning’s ability in automated public safety detection by roughly a 15% speed-up of inference using weight quantization.	The degree of accuracy reduction is contingent upon precision loss and the architecture of the network.
Kiran Ajmeera et al.^22^	Faster RCNN and YOLO	The paper presents a sophisticated surveillance system using deep learning techniques to find weapons and violence in camera data. Faster RCNN and YOLO help the model not only identify weapons but also classify their particular types, hence improving security and decision-making procedures in public settings by means of automated detection systems.	The paper investigates the conundrum of using Faster RCNN and SSD in real-time applications, stressing how Faster RCNN’s superior accuracy results in a sluggish frame rate, making it impractical for immediate use, while also noting their difficulty in distinguishing many instances of identical objects in an image, thus increasing the likelihood of false positives.
Singh et al.^23^	Faster R-CNN and YOLOv3	Leveraging the fast agility of YOLOv3 and the strong capabilities of Faster R-CNN, both trained on a varied dataset, this work presents a dynamic method to face mask identification. The findings highlight the important need for precise monitoring in public areas and provide tools for the next studies, thereby enabling developments in smart city projects and public health control.	In object identification models, speed and accuracy dance delicately; YOLOv3 moves forward for real-time application while Faster R-CNN takes its time for increased accuracy. But two-stage detectors’ processing load renders them unworkable in fast contexts, which limits their use in important situations like mobile technology and self-driving cars.
Santos et al.^24^	Faster R-CNN and YOLO	This work clarifies the difficulties in automatic weapon recognition, especially with relation to negative illumination situations and the difficulties in identifying small weapons. It emphasizes the need for using deep learning models such as Faster R-CNN and YOLO as well as the need for improved performance measures in challenging settings and the need for using both realistic and synthetic images for the best detection efficacy.	The accuracy of weapon detection is much reduced by dim lighting. Compact and hidden weapons reduce model efficacy; examples include knives and handguns. Current systems lose efficacy under real-time detection needs. Although it’s still a challenge, depending on quality data for model training is becoming less of a problem. Systems for automatic weapon identification fall short in demanding situations.
Pradnya More et al.^25^	CNNs and Bi-LSTMs	For quick violence and weapon identification in video streams, the novel method combines CNN with Bi-LSTM. While transfer learning increases performance even with limited data, VGG19 and LSTM achieve a remarkable 98% accuracy on the hockey dataset, and YOLOv8 excels across weapon picture datasets, hence enhancing detection power through spatial and temporal feature collection.	It underlines how impractical it is to count on people to sort through CCTV material in order to scale attempts at violence detection. According to the report, present monitoring systems typically focus on either violent events or weapon detection, rarely tackling both at once.
Uganya et al.^26^	FRCNN,Tiny YOLO	The work presents a deep learning surveillance system targeted at gun identification, hence improving law enforcement effectiveness. Especially in real-time crime detection, YOLO V3 shows exceptional performance, therefore underlining the predominance of handguns in robbery offenses and the effectiveness of the technology in evaluating complex surveillance patterns.	Creating a manual dataset for crime detection is a time-consuming and physically taxing task. Given various problems, real-time gun detection poses a difficulty. Detecting small objects in images gets difficult, particularly in pictures of like surroundings.
Ganesan et al.^27^	CNN	80% accuracy of anomaly prediction.	More challenging anomaly detection in certain environments.
Jain Dhiraj et al.^28^	FRCNN,YOLO v2	Accuracy is 95% in prediction	Limiting of true performance.

Vinay Gautam et al. developed a hybrid model of YOLO V5 and a convolutional neural network (CNN). This method has a high accuracy rate. Derbas et al.^29^ predicted voice, speech, and motion features via a multimodal approach to identify violence and non-violence. Using deep learning techniques can easily distinguish normal and abnormal activities and generate alerts or messages to law enforcement departments or security personnel in real time. This foresighted approach enables a fast response to potentially dangerous situations and helps relieve harmful situations. The physical and geographical outlier is user support. The invention of smart cities is not specifically addressed^30^. In this study, Narasimha Chary et al. used classifiers such as convolutional neural networks (CNN) and recurrent neural networks (RNN) for motion and object detection, achieving an accuracy rate of 80% in terms of prediction performance. The authors analyzed Philadelphia for anomaly detection and identified a trend. Machine learning technology, which uses techniques such as logistic regression, ordinal regression, decision trees, and k-nearest neighbors, achieves 60% accuracy^31,32^.

Tahreem Tahir et al. have presented an inquiry into the increasing of arms-related crimes, all in favor of exploring improved surveillance systems in providing safety interventions against gun violence. Sarfaraz Natha^33^, combining DenseNet 201 for spatial feature extraction and Bi-LSTM for temporal analysis−enhanced with a multi-attention mechanism−the CRVA model While our model specializes in real-time weapon detection with improved accuracy and efficiency, it targets general anomaly detection. Natha et al^34^, the SERAD model employs a stacked ensemble of VGG19, ResNet50, and InceptionV3 CNNs to detect and classify road anomalies in surveillance videos. Using spatiotemporal attention and ensemble CNNs such as CRVA and SERAD models shows good anomaly identification in surveillance videos. Building on these, our proposed work focuses especially on real-time weapon identification using a hybrid model that improves both detection precision and response speed.

Table 1 presents a collection of academic articles, detailing their methodologies, significant findings, and challenges in the domain of weapon detection. The progress achieved in weapon detection techniques represents a significant advancement in automated weapon detection, expanding the potential of this technology. This exploration lays the groundwork for more effective and dependable firearm detection systems, setting a benchmark for future inquiries and technological progress in the evolving field of automated weapon detection. The research studies focus on weapon use with deep learning models, putting focus on the balance between accuracy and real-time performance. YOLO-based models, with a focus on YOLOv7 and YOLOv8, demonstrated much higher accuracy, with YOLOv8 achieving a 90.1% mAP and beating previously mentioned models. YOLOv8 achieves a significant amount of inference speed enhancement by 15% in terms of weight quantization, indicating its future in real-time performance. Faster R-CNN and SSD models have also been contributing to firearm detection but ended up with challenges due to their speedy processing. Some studies combined several architectures, CNN with Bi-LSTM, aiming to enhance spatial-temporal analysis in video-based detection. The CNN-BiLSTM model attained an impressive accuracy of 98% on the hockey dataset. Similarly, studies combining Faster R-CNN and YOLO have rewarded important strides in firearm identification and classification towards public safety. However, since the frame rate was low, they suffered due to slower real-time applications compared to their faster R-CNN counterparts. Several limitations were enumerated by these studies. Detection accuracy was greatly affected by complex environments, lighting conditions, and occlusion problems. Small and concealed weapon detection, such as handguns and knives, was inherently hard. Real-time detection was compromised by speed-accuracy trade-offs, leaving models like YOLO to be really fast but at times sacrifice accuracy.

## Proposed methodology

**Fig. 1:**
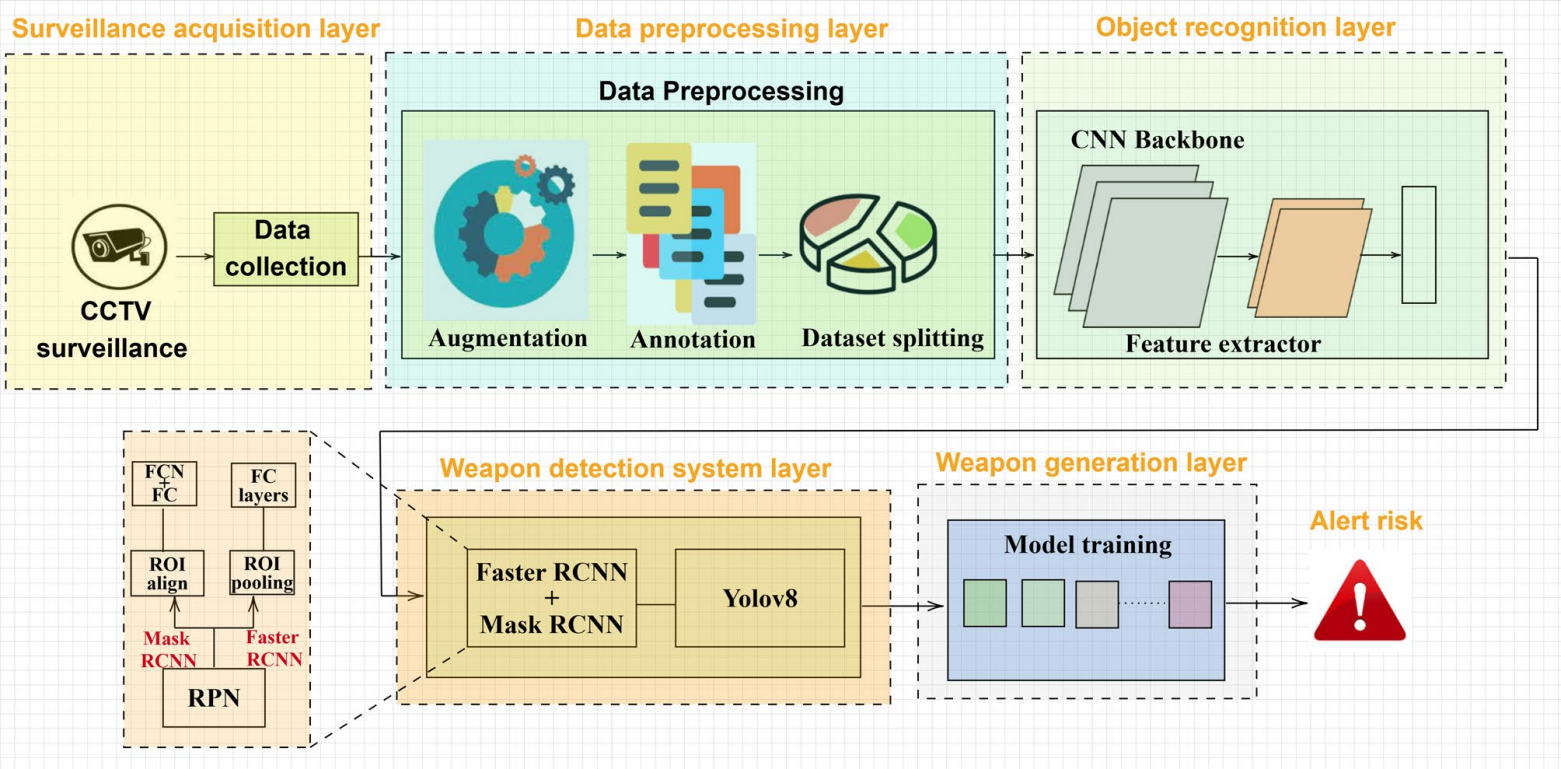
Architecture of the proposed real-time weapon detection system.

The multi-stage deep learning framework integrates object detection, classification, and instance segmentation approaches for the weapon detection system. It analyzes image frames extracted from CCTV video feed datasets in real time to identify and track weapons. It consists of multi-class classification tasks, segmentation for pixel-level object division, and real-time inference with high accuracy, precision, and rapid processing. The system proposed in this work is an advanced WDS (weapon detection system) for crime monitoring in real-time. Figure 1 presents the six layers of the proposed architecture.

### Surveillance acquisition layer

The layer captures real-time video feeds from CCTV cameras installed in public high-security areas. Furthermore, it includes a mechanism for collecting large-scale weapon datasets from CCTV footage and security databases to improve model training. This step will allow for constant monitoring for weapon detection and prevention, as the frame captured is the basis for the weapon detector system. The system will process the collected datasets frame by frame and then pass them into the preprocessing layer for enhancement, normalization, and resizing. The multiple camera sources support the system’s scalability for extensive video surveillance networks. This layer acts as the foundation of the WDS, enabling efficient and automated threat detection in real-time.

### Data preprocessing layer

The WDS preprocessing layer is to turn raw surveillance data into a structured format, make sure that everything is the same, improve image quality, and deal with real-world problems before the frames get to the detection layer. One of the primary challenges in surveillance footage is the inconsistent image sizes and proportions due to varying camera resolutions and angles; to overcome this, resize and standardize images to a fixed resolution for consistent processing. Low-light conditions and overexposure pose another challenge, making weapons harder to detect; to counter this, brightness enhancement and sharpening techniques refine image details, improve visibility, and feature clarity. Motion blur, compression artifacts, and environmental interference can all cause noise and distortions that make detection less accurate. Adaptive Gaussian Noise Injection can help with this by changing the amount of noise based on how clear an image is, mimicking flaws in the real world like motion blur and low resolution. Intelligent contrast and brightness adaptation improve visibility by using adaptive histogram equalization and gamma correction to make weapons stand out in different lighting conditions.

To improve model generalization across different perspectives, perspective-aware data augmentation introduces rotation to simulate weapons appearing at different angles, flipping involves mirroring images to introduce viewpoint diversity, and perspective transformations include occlusions and extreme camera tilts. Context-aware normalization changes the size of pixels based on changes in intensity. This makes sure that the model can handle big changes in lighting. For video surveillance, frames are selectively extracted using an adaptive sampling mechanism that takes into account changes in the scene and motion detection. This cuts down on unnecessary frames while keeping the important action moments. Annotation and labeling ensure accurate placement of the bounding boxes. Labels come in different formats, such as COCO, Pascal VOC, and YAML for YOLOv8, and segmentation masks help find the exact shape of weapons. Dynamic Dataset Splitting balances the dataset across training, validation, and testing by considering class diversity, ensuring better generalization. The labeled data is stored in YAML format for seamless multi-model incorporation to facilitate accurate detection and classification. The dataset is split into 70% training (model learning), 20% validation (hyper parameter tuning), and 10% testing (performance evaluation). This ensures that the training pipeline works and that the model performs well in new situations. These new improvements make the preprocessing layer much better at adapting, being robust, and finding weapons accurately. This makes it possible to reliably identify weapons in real-world surveillance settings.

### Object recognition layer

**Fig. 2:**
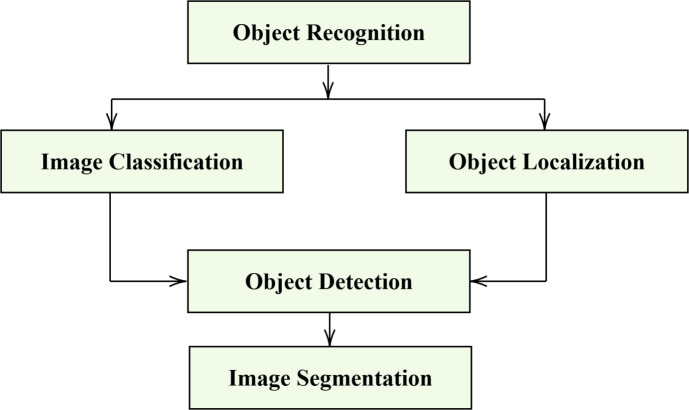
Object detection process.

Advanced network techniques analyze and identify specific objects within both images and videos, a process known as object recognition as shown in Fig. 2, to provide a precise interpretation of a given image. The process begins with object detection based on predefined parameters, followed by high-accuracy object recognition to ensure clear classification. Furthermore, advanced programs improve the system by allowing object tagging for faster identification. They methodically categorize images in the context of weapon detection into two categories: object recognition and object detection, ensuring a robust and accurate classification system^35^. It minimizes the model size and loss functions to enhance object recognition and retraining parameters. This method reproduces a network model with excellent accuracy and rapid detection speed^36^.

Object detection becomes significantly more efficient with the integration of modern deep learning algorithms. These models leverage neural networks and deep learning, marking the next evolution in machine learning. Template matching, color-based detection, active and passive recognition, and shape-based analysis are just some of the advanced techniques that advanced object recognition algorithms used to accurately and reliably identify objects. An object recognition model is employed as a backbone of the convolutional neural network (CNN), and MobileNetV3 is a state-of-the-art neural network. It solves it by providing a lightweight and efficient model that can classify objects with limited training data.Employing prominent feature extraction methods like depth-wise separable convolutions enables faster yet effective object localization and detection for real-time applications without compromising accuracy.

**Fig. 3:**
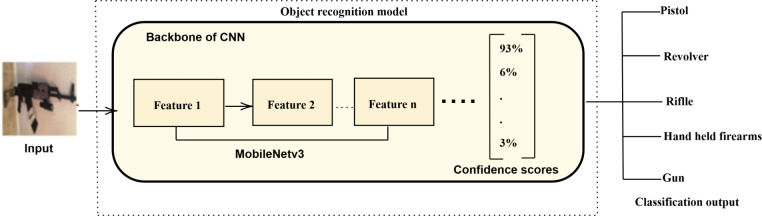
Weapon feature detection.

This means the output has a high probability the object in the picture is accurate, and the rest of the output probabilities are low or can be avoided. Figure 3 illustrates the system getting a weapon image as an input, processing it through preprocessing stages, and extracting features using MobileNetV3 layers. At last, output of confidence scores for different weapon classes, with the percentages indicating the model’s confidence in each classification.

**Fig. 4:**
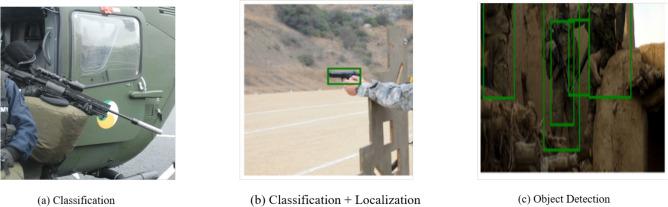
Weapon feature detection.

When classifying an image, it takes the image as input^37^, and its classification label is output on the basis of a set of criteria, such as probability, loss, accuracy, etc. Object localization involves detecting the exact location of a particular image in place and using a bounding box to represent it. After receiving an image as input, the system outputs the bounding box’s location in terms of position, height, and width. It achieves object detection through a combination of image categorization and object localization as shown in Fig. 4. An image input generates one or more bounding boxes with the class label angled toward it. Object segmentation is an advanced version of object detection that uses pixel-wise masks created for each object in the image to indicate its appearance. That approach is more familiar in Mask R-CNN.

### Weapon detection system layer

In surveillance situations, the WDS uses a multi-aspect solution that combines the power of FMR-CNN and YOLOv8 to identify weapons from start to finish. The Faster R-CNN model gives accurate classification and localization of detected weapons through improved bounding boxes. Mask R-CNN works in the background to create detailed segmentation masks of weapon shapes. This allows for accurate object boundary detection and lowers false positives through shape analysis. Meanwhile, YOLOv8 quickly scans video feeds at 30+ frames per second, flagging any possible weapon presence in real time.

#### Detection network using Faster R-CNN

**Fig. 5:**
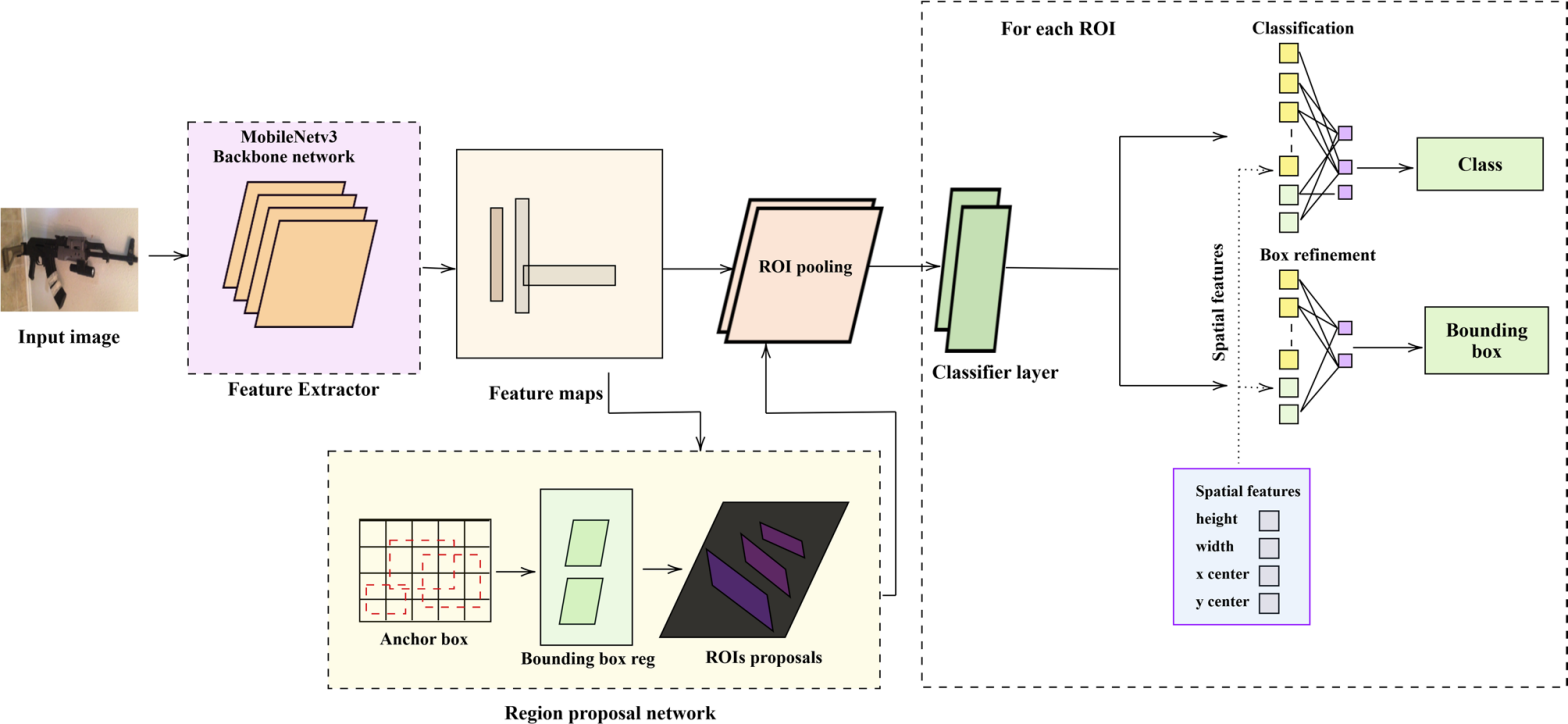
Faster RCNN system model.

The faster region-based convolutional neural network state-of-the-art algorithm is used to detect different objects, especially weapons, in pictures. It is the most powerful tool of deep learning methods and is based on an object detection framework that has been used for a range of applications, such as security and surveillance. The deep learning-based object detection structure shown in Fig. 5 is known for being accurate and being able to find objects quickly and correctly. The object detection works are divided into two categories: the region proposal network and object classification. In the study, Changqing Cao et al.^38^ A faster RCNN with an atrous RPN (Region Proposal Network) is a better way to detect things that uses dilated convolutions and ”holes” (atrous) in the kernel. This architecture aims to expand the receptive field while maintaining spatial resolution. This network processes features at multiple scales continuously by applying varying dilation rates, making it particularly effective for weapon detection, as it can better handle varying sizes, distances, and orientations while maintaining high accuracy and computational efficiency. It examines the object background at different scales by sliding an organization of filters that are applied to the final convolution feature map, increasing the dilation rates.

Region Proposal Network (RPN) elements combined with a model that efficiently balances precision and speed of inference. It helps to accurately analyze the small items inside a bigger picture. This approach is especially important for real-time CCTV monitoring since quick detection and reporting help security to react to possible hazards. Pre-trained weights from the Gunmen dataset^37^, a high-volume, extensively used benchmark dataset, are used in fine-tuning to improve model performance. Leveraging past knowledge on a large collection of photos guarantees enhanced accuracy, resilience, and adaptability in identifying criminal activity inside surveillance environments, therefore providing the main advantage of this method.

Convolutional Neural Networks (CNNs) are the most popular way to extract features, which is the most important step toward learning representations that are organized in a hierarchy. They are the building blocks of hierarchical representation learning. In the process of convolution, several layers are refining input images. In the first layers, we can see the concept of basic low-level elements like edges, textures, and gradients that are captured. The deeper layers implement the high-level semantic part of the elements found in the image. That layer also keeps the important geometrical and spatial data, which helps with detection tasks that need to be accurate and reliable.

The creation of hierarchical features permits weapon analysis to run perfectly because it includes multi-scale details, edges, and context dependencies. This is allowed by the use of the MobileNetV3 integration with depthwise separable convolution. It makes parameters simpler and reduces the time it takes to draw conclusions. Adding Neural Architecture Search (NAS) to the MobileNetV3 architecture also makes the network automatically optimize its topology. This makes the network balance latency, accuracy, and edge applications. With the squeeze-and-excitation SE module, the datasets adjust their feature activations dynamically to decrease the repeating data and defend weapon-related areas within an image. Because of these improvements, MobileNetV3 is excellent at finding things and utilizing computer power efficiently, making it ideal for real-time security uses. It receives pre-trained convolutional feature maps composed of spatial and contextual tokens. The RPN gets its value from the Faster R-CNN’s in-between layers. To quickly produce high-confidence scores by generating the most accurate bounding regions. This model is a joint combination of those two streams that is supposed to be the best choice for the purposes of real-time security surveillance as well as threat detection. The RPN region proposals for consecutive detection of objects. It works by sliding a small network, such as a CNN, over the convolution element maps formed by the backbone network. These proposal networks are probably bounding boxes that may contain objects of interest such as weapons, representing Eqs. (1) and (2)

1 $$\begin{aligned} p_0(r)= & \text {Probability of object for region } r \end{aligned}$$

2 $$\begin{aligned} \Delta (x, y, w, h)(r)= & \text {Bounding box offsets for region } r \end{aligned}$$

Anchor boxes at different scales and aspect ratios can be used to efficiently capture weapon regions, especially those capable of detecting weapons. Let A denote the set of anchor boxes, each box systematically by its mid-coordinates, width, and height. The convolution sliding window over feature maps produces scores and bounding box offsets for each anchor. The dimensions of the feature maps are W × H and K anchor boxes, and the output scores of bounding box offsets are substituted as and. Bounding box regression predicts adjustments to the anchor boxes to improve the fit of the objects. The adjusted bounding box is equal to the loss function of the RPN, which is trained via a combination of classification and regression losses, typically binary cross-entropy loss for object scores and smooth L1 loss for bounding box offsets in Eq. (3).

3 $$\begin{aligned} L_{\text {cls}} = -\sum _i \left[ p_i^* \log p_i + \left( 1 - p_i^*\right) \log (1 - p_i) \right] \end{aligned}$$

$p_i^*$ - ground truth label (1 for an object, 0 for background). $p_i$ - predicted probability for an object

Region of interest (ROI) pooling is designed to extract fixed-size feature maps from variable-sized regions of interest. The mechanism of ROI pooling is a two-step process involving quantization and max pooling. Quantization involves mapping the coordinates of the variable-sized ROIs to a fixed-size grid. The RoI coordinates are for the upper-left corner and for the lower-right corner, and the width w and height h of the RoI are calculated as in Eqs. (4) and (5).

4 $$\begin{aligned} w&= x_2 - x_1 \end{aligned}$$

5 $$\begin{aligned} h&= y_2 - y_1 \end{aligned}$$

The grid division divides the RoI into a fixed number of bins, typically H$\times$W, where H represents the number of vertical bins (height). W represents the number of horizontal bins (width). Each bin has the following dimensions, as in Eqs. (6) and (7).

6 $$\begin{aligned} \text {bin width}&= \frac{w}{W} \end{aligned}$$

7 $$\begin{aligned} \text {bin height}&= \frac{h}{H} \end{aligned}$$

The range of pixels from the original feature map that fall within this bin. This involves converting the continuous coordinates into discrete bin indices. Let us denote the feature map dimensions as and as in Eqs. (8) and (9). For bin (i, j), the ranges of the coordinates in the feature map are as follows in Eqs. (10) and (11).

8 $$\begin{aligned} x_{\text {start}}&= x_1 + \left( \frac{j \cdot w}{W} \right) \end{aligned}$$

9 $$\begin{aligned} x_{\text {end}}&= x_1 + \left( \frac{(j+1) \cdot w}{W} \right) \end{aligned}$$

10 $$\begin{aligned} y_{\text {start}}&= y_1 + \left( \frac{i \cdot h}{H} \right) \end{aligned}$$

11 $$\begin{aligned} y_{\text {end}}&= y_1 + \left( \frac{(i+1) \cdot h}{H} \right) \end{aligned}$$

Max pooling is applied within each bin to down sample the region and extract the most important feature. This is particularly useful for detecting weapons, which might occupy small parts of the RoIs. For each bin(i,j) in Eq. (12), after quantization, there is a sub region of the element map. Max pooling selects the high value within this sub region.

12 $$\begin{aligned} \text {Pooled value}_{(i,j)} = \max \left\{ \text {feature}\_\text {map}[y,x] \,|\, x \in [x_{\text {start}}, x_{\text {end}}],\ y \in [y_{\text {start}}, y_{\text {end}}] \right\} \end{aligned}$$

In classification and bounding box regression, localization and identification of weapons in images are involved, as Eq. (13). The classification branch predicts the probability of each proposed region containing a weapon. It is achieved by passing the extracted feature maps from the ROI pooling layer through a completely connected layer continued by a Softmax activation function. The probability distributions over various classes, especially the classes corresponding to weapons.

13 $$\begin{aligned} P(\text {weapon} \mid \text {region}) = \text {softmax}(w_c \cdot \text {features}) \end{aligned}$$

where, $P(\text {weapon} \mid \text {region})$ is the probability of the region containing a weapon. $w_c$ represents the weight matrix of the fully connected layer. *features* are the extracted feature maps from the ROI pooling layer.

The substantive locations of the weapons within the picture are used to refine the coordinates of the proposed bounding boxes. This is accomplished by predicting adjustments (offsets) to the coordinates of the initially proposed bounding boxes. The regression boxed features compute a likelihood vector of the equation regression, where the mid-coordinates of the bounding box are proposed. Pw and Ph are proposed tries to the width and height of the bounding box. The refined bounding box coordinates are calculated as follows in Eq. (14):

14 $$\begin{aligned} (x_{\text {refined}},\ y_{\text {refined}},\ w_{\text {refined}},\ h_{\text {refined}}) = \left( x + p_x \cdot w,\ y + p_y \cdot h,\ w \cdot \exp (p_w),\ h \cdot \exp (p_h) \right) \end{aligned}$$

where, (*x*, *y*, *w*, *h*) are the coordinates of the proposed bounding box. $(x_{\text {refined}},\ y_{\text {refined}},\ w_{\text {refined}},\ h_{\text {refined}})$ are the refined bounding box coordinates.

To encapsulate the entire weapon detection process using Faster R-CNN can be expressed as in Eqs. (15), (16) and (17) follows:

15 $$\begin{aligned} \hat{B},\ \hat{C} = \arg \max \sum _{i=1}^{N} \left[ P(C_i \mid F_R) + \lambda \cdot {\mathcal {L}}_{\text {reg}}(B_i, B_i^*) \right] \end{aligned}$$

where, $\hat{B},\ \hat{C}$: Predicted bounding boxes and weapon labels. *N*: Total number of region proposals. $P(C_i \mid F_R)$: Probability of class $C_i$ (weapon or non-weapon) given the extracted feature map $F_R$, computed using softmax.

16 $$\begin{aligned} P(C_i \mid F_R)= & \frac{e^{W_c \cdot F_R}}{\sum _j e^{W_c \cdot F_R}} \end{aligned}$$

${L}_{\text {reg}}(B_i, B_i^*)$: Bounding box regression loss (Smooth L1 loss) between predicted $B_i$ and ground truth $B_i^*$

17 $$\begin{aligned} \mathcal {L}_{\text {reg}}(B_i, B_i^*) = \sum _{j \in \{x, y, w, h\}} \text {smooth}_{L1}\left( t_{ij} - t_{ij}^*\right) \end{aligned}$$

$\lambda$: Balancing factor for classification and regression loss.

This formula optimizes both classification (weapon detection probability) and bounding box localization, ensuring that the detected weapons are correctly identified and precisely localized. Faster R-CNN, combined with MobileNetV3, provides an effective weapon detection framework. By leveraging feature extraction, region proposals, and bounding box regression, this model achieves high accuracy and computational efficiency, making it ideal for real-time security applications.

#### Detection network using Mask RCNN

**Fig. 6:**
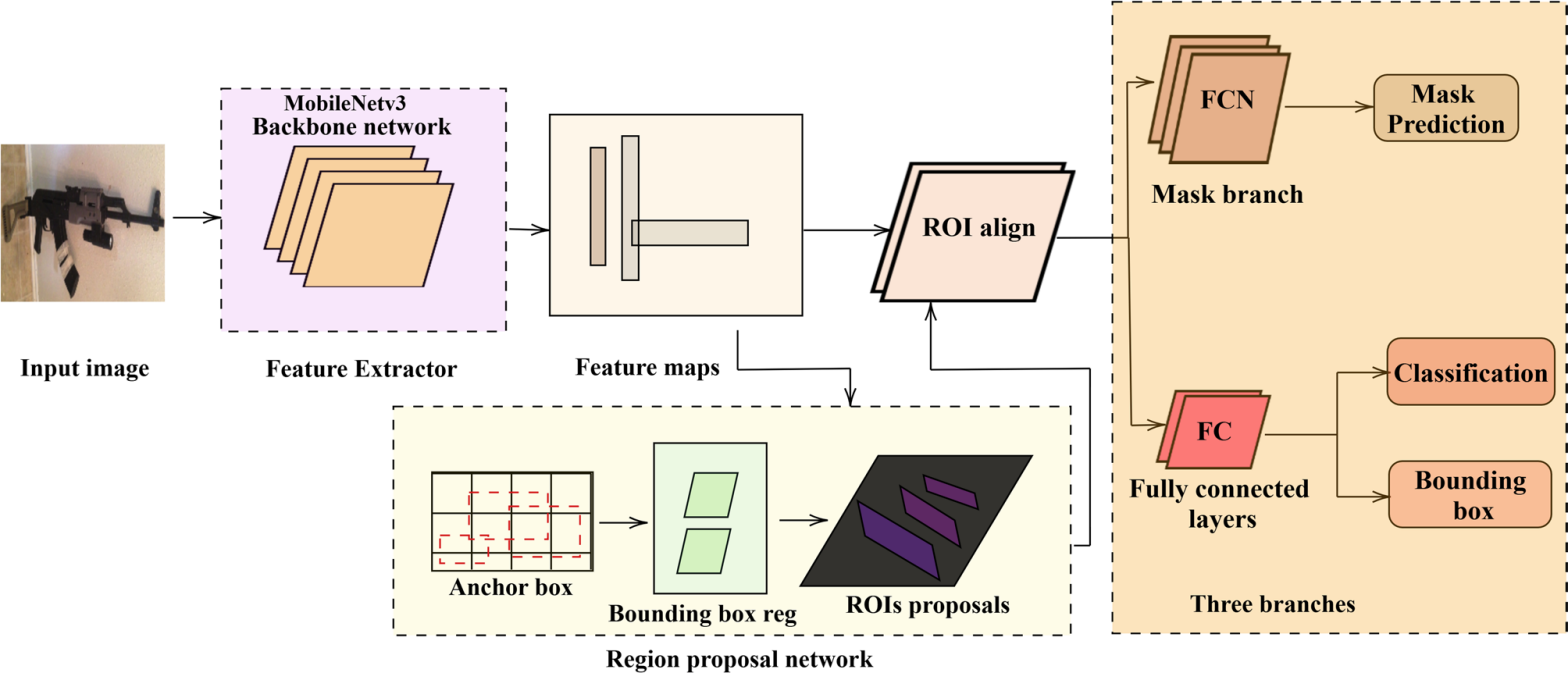
Mask RCNN system model.

The Mask R-CNN extends the Faster R-CNN by joining an additional branch for analyzing segmentation masks for each detected object. It focuses mainly on performing object detection, classification, and segmentation. Segmentation plays an important role in weapon detection by accurately characterizing the region of interest (ROI) in a picture. The image segmentation technique is used to classify the pixels in an image into determined categories to enable clear localization of weapons. By segmenting the picture into semantic regions such as “weapons” and “background.” In Fig. 6, the backbone network in Mask R-CNN for weapon detection, a CNN like MobileNetv3, is responsible for expressing rich, hierarchical features from the input image^37^. In this work, focus on semantic segmentation via the Mask R-CNN algorithm. For the Mask R-CNN with semantic segmentation, the mathematical formulation combines the object detection capabilities of the Mask R-CNN with pixel-level semantic segmentation.

The ROI layer extracts features from the region of interest (ROI) in the feature map F, and the results can be processed further. This technique helps in accurately extracting feature maps for analyzing objects from pictures. It is important for security purposes, especially in detecting criminal activities, as in Eqs. (18) and (19). The mathematical formula for the ROI align operation in a CNN can be split into steps involved in bilinear interpolation to illustrate the values at non-integers on a feature map. The values at (a, b) are as follows, as in Eqs. (20) and (21):

(A) Interpolate with respect to a:
  18 $$\begin{aligned} f(a, b_1)= & f(a_1, b_1)(a_2 - a) + f(a_2, b_1)(a - a_1) \end{aligned}$$
  19 $$\begin{aligned} f(a, b_2)= & f(a_1, b_2)(a_2 - a) + f(a_2, b_2)(a - a_1) \end{aligned}$$
(B) Interpolate along y:
  20 $$\begin{aligned} f(a, b) = f(a, b_1)(b_2 - b) + f(a, b_2)(b - b_1) \end{aligned}$$
  Using a weighted average of the four nearest known values, bilinear interpolation, a resampling technique, estimates values at intermediate positions in a 2D grid. To guarantee seamless transitions, it linearly interpolates first in one direction (x, y) and then in the other. Image processing and computer vision both extensively apply this method for resizing and image transformation while maintaining spatial consistency. Overall, the mathematical expression for bilinear interpolation, f (x, y), is written as
  21 $$\begin{aligned} F(a, b) = \left[ f(a_1, b_1)(a_2 - a) + f(a_2, b_1)(a - a_1) \right] (b_2 - b) + \left[ f(a_1, b_2)(a_2 - a) + f(a_2, b_2)(a - a_1) \right] (b - b_1) \end{aligned}$$
  Region Proposal $r \in R$, the classification scores $C_c$ and bounding box coordinates $B_r$ are recognized by the object detection head. $C_c(r)$ is the probability of class for region *r*, and $B_r$ represents the bounding box coordinates for region *r*. The semantic segmentation network (SSN) analyzes the pixel-wise segmentation mask *M* for a given input image *I*. *M*(*x*, *y*, *k*) denotes the probability of pixel (*x*, *y*) belonging to class *k*. The segmentation mask $S_c$ combined with each class *c* is pulled from the pixel-wise segmentation mask *M*. This provides fine-grained spatial information with respect to object boundaries and interior regions, which can be used to enhance object detection productivity. Region Proposal: The classification scores Cc and bounding box coordinates are recognized by the object detection head. The semantic segmentation network (SSN) analysis pixel-wise segmentation mask (M) for a given input image I.M(x, y, k) = Probability of pixel (x, y) attached to class k. The segmentation mask Sc combined with each class c is pulled from the pixel-wise segmentation mask M. This provides fine-grained spatial information with respect to object boundaries and interior regions, which can be used to enhance the object detection productivity.

Mask forecasting involving the mask RCNN improves the detection process by generating clear segmentation masks for each identified weapon. The next step in the network’s progress is to classify regions of interest (RoIs). The mask prediction branch uses RoIs to get binary masks that show the exact shape and boundaries of the weapons. This stage helps with pixel-level accuracy in recognizing weapons; it enhances the ability to distinguish between various types of weapons and provides elaborate information about their area and location within the image. As shown in Eq. (22), these exact calculations are very important for security surveillance and automated threat detection because they look at the shape and features of weapons, make it easy to see what kinds of weapons are out there, and make sure that responses are accurate and effective.

22 $$\begin{aligned} \hat{B}, \hat{C}, \hat{M} = \arg \max \sum _{i=1}^{N} \left[ P(C_i \mid F_R) + \lambda _1 \cdot \mathcal {L}_{\text {reg}}(B_i, B_i^*) \right] + \lambda _2 \cdot \mathcal {L}_{\text {mask}}(M_i, M_i^*) \end{aligned}$$

where, *M*: Segmentation mask $\mathcal {L}_{\text {mask}}(M_i, M_i^*)$: Binary cross-entropy loss for the segmentation mask $\lambda _1$ and $\lambda _2$: Weighting factors for balancing classification, localization, and segmentation losses. This formula integrates classification, bounding box regression, and mask prediction into a single, optimized loss function, making Mask R-CNN superior for weapon detection in security applications.

#### Integration of the Faster RCNN with the Mask RCNN

Faster R-CNN has trouble distinguishing overlapping weapons in cluttered or obstructed situations due to inadequate object segmentation. It’s slower than YOLO, and multi-stage processing makes computing expensive. It also suffers from localization problems and finds small or partially concealed weapons challenging. On the other hand, Mask R-CNN has a high computational load due to its segmentation branch, making real-time detection challenging. It also requires well-annotated datasets for accurate segmentation, and poor labeling can lead to detection failures. To get around these problems while combining FMR-CNN as in Eq. (23), improve weapon detection by combining precise segmentation with bounding box detection. ROI Align eliminates misalignment issues, improving localization accuracy. A shared CNN backbone optimizes computation, reducing processing overhead. Segmentation masks refine small or occluded object detection, ensuring better distinction in crowded scenes. Faster R-CNN filters irrelevant objects before segmentation, reducing Mask R-CNN’s computational cost and making the system efficient and accurate.

23 $$\begin{aligned} L_{\text {RPN}} = L_{\text {cls}} + \lambda L_{\text {reg}} \end{aligned}$$

where, $L_{\text {cls}}$ - classification loss. $L_{\text {reg}}$ - regression loss. $\lambda$ - weighting factor

**Significance:**

- The system can simultaneously classify weapons and generate pixel-wise masks, leading to better decision-making in surveillance applications.
- The integrated model triggers real-time alerts to law enforcement, allowing preventive action.
- Mask R-CNN’s segmentation masks help detect hidden weapons, which Faster R-CNN alone might miss.
- This is especially useful in crowded places where people may conceal weapons.
- It reduces false positives and false negatives, making the system more reliable in real-world applications.
- A novel deep learning-based approach that outperforms traditional models.

#### YOLOv8

The proposal consists of a YOLOv8-based approach to weapon detection in smart cities. The intentions of the approach are to mitigate the pre-defined challenges and limitations present in previous YOLO versions while also enhancing accuracy and performance for real-time analysis, flexibility, reducing false positives, and reducing cost of operation.The YOLOv8 algorithm detects objects exactly like person or object frames and operates in parallel with classification and boundary frame regression, which is highly accurate^39^. The attention module for the feature extraction is used as the backbone network of the C2F structure^40^, and the detection of objects works as a regression problem, utilizing convolution layers, pooling layers, and fully connected layers to analyze the locations of objects and classes^41^. Different cutting-edge technologies have been incorporated, improving the accuracy and robustness of detection^42,43^. YOLO versions typically evolve, and the kinds of sub-algorithms and features that could be expected to be emphasized or newly introduced in a hypothetical YOLOv8, particularly for applications such as weapon detection. It integrates with feature pyramid networks (FPNs) to improve the detection of objects. FPNs represent a significant promotion in object detection models such as YOLO V8, offering increased multiscale feature incorporation, hierarchical fusion, adaptive refinement, efficient computation, and seamless integration. These enhancements mainly contributed to enhanced object detection performance, particularly in detecting objects at various scales, including weapons across diverse surveillance environments. Utilization of AutoML and neural architecture search (NAS) and knowledge distillation are incorporated into the technique of the advanced training process. In this work, the use of an ensemble of various detection models, including different versions of YOLOv8 or two-stage detectors such as Faster R-CNN, Mask R-CNN, and DeepCNN, to produce a vote or average out of the detection results can improve reliability and liability. The use of ensemble methods with YOLOv8 to detect a weapon is as follows in Eq. (24): 1. High Accuracy 2. Robustness 3. Confidence Estimation. The Bayesian inference for confidence estimation to predict the models involves calculating the posterior probability of the presence of a weapon given the observed evidence from all models:

24 $$\begin{aligned} P(\text {weapon observation})&= \frac{P(\text {Observation} \mid \text {Weapon}) \cdot P(\text {Weapon})}{P(\text {Observation})} \end{aligned}$$

EfficientNet is part of a convolution neural network (CNN) designed to achieve better efficiency and accuracy by balancing depth, width, and resolution. It introduces a compound scaling method that uniformly scales all three dimensions of the network: depth (d), width (w), and resolution(r) via a set of fixed scaling coefficients. The scaling is determined Eqs. (25)–(27):

25 $$\begin{aligned} d&= \alpha \cdot \varnothing \end{aligned}$$

26 $$\begin{aligned} w&= \beta \cdot \varnothing \end{aligned}$$

27 $$\begin{aligned} r&= \gamma \cdot \varnothing \end{aligned}$$

Here $\varnothing$ controls the scale of resources; a, b, and g determine how much each dimension (depth, width, and resolution) could increase along with a given increase in resources f in Eq. (28). A greater advantage in capturing finer details of smaller objects via these parameters can be customized based on the typical size, shape, and appearance of weapons in surveillance footage. The number of parameters and the computational cost of efficient nets measured as FLOP (Floating Point Operations) can be acquired more efficiently than other ResNet or DenseNet methods, especially those used to detect weapons.

28 $$\begin{aligned} C = \text {FLOPs} = \mathcal {O}(d \times w^2, r^2) \end{aligned}$$

Where O is a function of the number of operations on the basis of depth, width, and resolution and helps to manage complexity effectively, increasing both accuracy and speed. Large-scale image datasets can be pretrained by EfficientNet, such as ImageNet. These pretrained images could be converted into other objects through fine-tuning. The pretrained EfficientNet model, along with weights and the new task model, is fine-tuned such that these weights are slightly suitable for where they are in Eq. (29).

29 $$\begin{aligned} W_{\text {new}} = W_{\text {pre}} + \Delta W \end{aligned}$$

Here, $W_{\text {pre}}$ - Pretrained EfficientNet model along with weights. $W_{\text {new}}$ - Fine-tuned model for the new task. $\Delta W$ - Change in weights

Studied new task-specific data to recognize weapon characteristics. MobileNetV3 performs well in applications such as the detection of weapons, where speed, efficiency, and deployment on mobile or edge devices are requisite. It uses a Standard Convolution Cost (SCC) in Eq. (30), and Depth wise separable convolutions (DSC) in Eqs. (31) and (32), which are factorized are divided into two layers: 1. Depthwise convolution and 2. Pointwise convolution. This method reduces the computational cost and the number of parameters in Eq. (33).

30 $$\begin{aligned} C_{\text {std}}&= k \times k \times D_{\text {in}} \times D_{\text {out}} \times w \times H \end{aligned}$$

Depth wise separable Convolution Cost:

31 $$\begin{aligned} depth wise\,\,convolution: C_{\text {dw}}&= k \times k \times D_{\text {in}} \times w \times H ,\end{aligned}$$

32 $$\begin{aligned} Pointwise\,\,convolution:C_{\text {pw}}&= D_{\text {in}} \times D_{\text {out}} \times w \times H \end{aligned}$$

33 $$\begin{aligned} Total\,\,cost:C_{\text {ds}}&= C_{\text {dw}} + C_{\text {pw}} , \end{aligned}$$

where, *k* is the kernel size, $D_{\text {in}}$ and $D_{\text {out}}$ are the numbers of input and output channels *w* and *H* are the width and height of the input map, respectively,

**Fig. 7:**
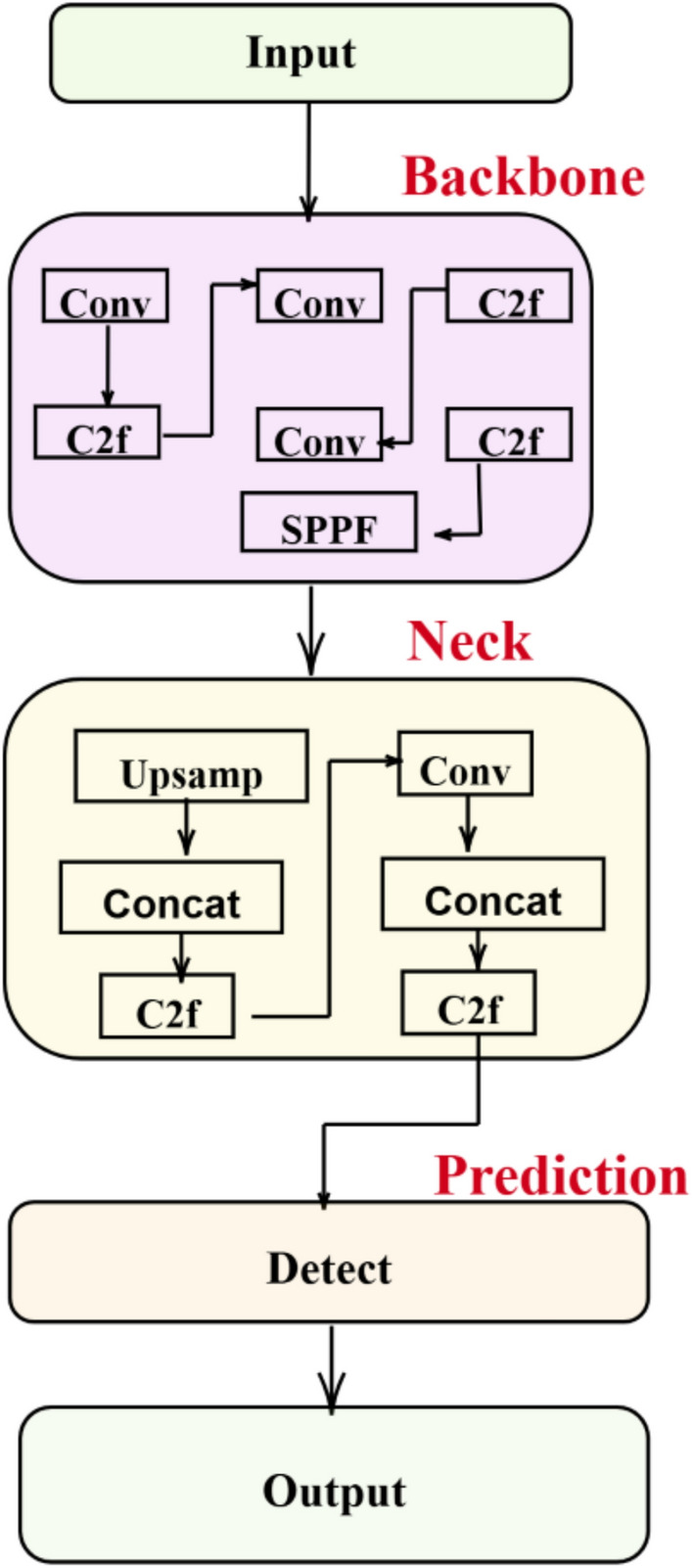
YOLOv8 model.

By reducing the computational overload, MobileNetv3 can process images as quickly, which is vital for weapon detection. The inverted residual block feature carries a lightweight as the important computational component inside a residual block, which detects distinct weapon features such as outlines or specific parts, as shown in Fig. 7. The preserved volume of information, which is critical for explained feature extraction, is required for detecting small objects or partially dark weapons via linear bottlenecks. The implementation of these methods within the YOLO-based weapon detection framework reduces the computational load, and these features of linear bottlenecks and depth-wise separable convolutions ensure that even small or partially visible weapons can be detected effectively, improving the overall security and responsiveness of surveillance systems. Hence, by incorporating EfficientNet or MobileNetV3, YOLOv8 could achieve swift inference times and high accuracy, making it useful for object detection, such as weapon detection.

Non-maximum suppression is a vital post-processing technique that processes detection to suppress overlapping bounding boxes with lower confidence scores. In this way, non-maximum suppression replaces multiple redundant and low-confidence-detected bounding boxes with a small and more accurate subset of the set of detected objects. Moreover, non-maximum suppression can be formulated as follows. Non-maximum suppression is an algorithm that iterates over all proposed bounding boxes and suppresses those that have a high overlap (IOU−Intersection over Union) with other bounding boxes of higher confidence. The IOU is a proportion of the overlap between two given bounding boxes. It equals the area of the intersection of these two bounding boxes divided by the area of the union of these two bounding boxes. The area is predicted, and ground truth bounding boxes are combined. It represents the region where both bounding boxes cover the same object. The total area is covered by both the predicted and ground truth bounding boxes. It includes the overlapping area as well as the areas covered exclusively by each bounding box in Eq. (34),

34 $$\begin{aligned} \text {IOU} = \frac{\text {Area of Intersection}}{\text {Area of Union}} , \end{aligned}$$

Each bounding box is associated with a confidence score, which represents the model’s belief that it contains an object of interest, i.e., a weapon. The NMS prioritizes bounding boxes with higher confidence, which is considered the confidence score. During NMS, bounding boxes are then sorted in descending order of their confidence scores. An IOU is computed between the bounding box of interest and all the bounding boxes that follow it in the list. An IOU check is then conducted, where if the overlap is more than a certain IOU threshold, NMS is typically empirically chosen to be 0.5 or 0.7. Suppression in Eq. (35): Then, the box that has a lower confidence is suppressed.

35 $$\begin{aligned} \text {Suppress}(\text {box}_i) = {\left\{ \begin{array}{ll} 1, & \text {if } \text {IOU}(\text {box}_i, \text {box}_j) > \text {IOU Threshold} \\ 0, & \text {otherwise} \end{array}\right. } , \end{aligned}$$

After the completion of NMS, the output detection contained in all of the bounding boxes is the final detection of objects, overwhelmingly a weapon identified in the image. The output is generally associated with the scores assigned to each urgency displayed on the source image, as in Eq. (36).

36 $$\begin{aligned} \hat{B},\ \hat{C},\ \hat{P} = \arg \max \sum _{i=1}^{S \times S \times A} \left[ 1_{\{obj\}} \left( \lambda _{\text {coord}} \mathcal {L}_{\text {coord}}(B_i, B_i^*) \right) + \mathcal {L}_{\text {cls}}(C_i, C_i^*) \right] , \end{aligned}$$

where, $\hat{P}$: Confidence score. $S \times S$: Number of grid cells. *A*: Number of anchors per grid cell. 1$_{\{obj\}}$: Indicator function (1 if object exists in the grid cell, 0 otherwise). The network simultaneously predicts bounding boxes, class labels, and confidence scores, enabling real-time of weapon detection.

#### Hybrid model (FMR-CNN + yolov8)

FMR-CNN enhances spatial feature extraction and segmentation, which complements YOLOv8 by enabling faster and more precise real-time weapon detection. This synergy improves robustness under occlusion and low-resolution conditions. After the CSPDarknet53 backbone collects important visual elements from the input image, the procedure proceeds with FMR-CNN’s refinement module, which boosts weak signals and reduces background noise−qualities absolutely vital for the detection of small and obscured weapons. After that, the improved feature maps are processed via YOLOv8’s detection head to produce initial bounding box suggestions that are further modified by FMR-CNN’s localization module, therefore guaranteeing exact alignment with actual weapon bounds. By dynamically balancing YOLOv8’s quick estimations with FMR-CNN’s deep feature validation, a confidence scoring system lowers false detections and raises classification accuracy. At last, the system generates quite accurate weapon classifications with ideal bounding boxes, obtaining 90.1 AP while preserving almost real-time inference at 9.2 FPS. Combining the best of speed and accuracy, this hybrid technique beats stand-alone models and is therefore the perfect choice for real-time security and monitoring uses, as in Eq. (37).

37 $$\begin{aligned} CL_{\text {Hybrid}} = \lambda _1 L_{\text {YOLO}} + \lambda _2 L_{\text {FMR}} + \lambda _3 L_{\text {IoU}} , \end{aligned}$$

where, $L_{\text {YOLO}}$ is the YOLOv8 loss (classification + regression). $L_{\text {FMR}}$ is the FMR-CNN feature refinement loss. $L_{\text {IoU}}$ penalizes overlapping detections. Contributions of the hybrid model (FMR-CNN + YOLO8)

Through integration of FMR-CNN’s multi-scale feature map enhancement, the hybrid model enhances small-object detection.

- Innovative fusion algorithm combining high-precision detections with real-time processing.
- The model dynamically balances confidence ratings, hence lowering false positives while preserving high detection speed.
- More accurate than YOLOv8 alone, it achieves near-real-time FPS (9.2) while increasing AP to 90.1; therefore, it remains computationally efficient.
- For practical security uses, this hybrid model is unique in its capacity to improve YOLOv8’s speed with FMR-CNN’s improved detection.

#### Weapon detection algorithm

The WDS algorithm uses computer vision techniques to detect weapons in CCTV footage. To do this, the WDS algorithm, as outlined in Algorithm 1, makes use of a pre-trained object detection model that was trained on a large dataset containing images of weapons and behavioral activity images. The output is detected objects given the input of dataset of video frames, classes such as “gun”,” rifle”, “pistol”, “revolver”, and “short handheld firearm.” Each video frame is iterated through by the algorithm, and each process can confirm before sending the message to the multi-model for object detection. The algorithm sounds an alert and informs the responsible authority departments if a weapon class is found. The weapon detection algorithm tool that facilitates fast and efficient reactions to possible weapon threats.

### Weapon generation layer

Accurate classification in the Weapon Generation Layer: the objects detected from the Weapon Detection System (WDS) are refined through a structured model training-based approach. After the Faster R-CNN detection model, Mask R-CNN, and YOLOv8 models detect a weapon, its region is cropped, and its spatial and contextual information is preserved by passing it through a feature pyramid network (FPN)-based deep feature extractor. Thereafter, these refined features are passed through fully connected layers (FCN) and class-specific classifiers, marking each weapon detection with a confidence score that remarks on accuracy in the learned feature representations. For higher-level bounding box localization, the system uses a hybrid decision fusion approach, which combines outputs from Faster R-CNN, segmentation-based Mask R-CNN, and real-time detection-friendly YOLOv8. Now the system can determine the contribution weight of the models automatically using the operational confidence-weighted averaging (CWA) mechanism. Detections that models agree on more receive stronger classification trust, while detecting lower-agreeing patterns receives lower confidence. In real-life situations, this technique can help reduce false positive detection of weapon classes while having their classification confidence hard-threshold to 0.75 instead of 0.7. Utilizing learned feature correlation, the model categorizes the identified object as either a handgun (pistol, revolver), rifle (assault rifle, sniper), or shotgun (pump-action, tactical) depending on the observed class patterns.At last, the confirmed weapon categories are saved, and a risk evaluation is performed before raising an alert, which guarantees a model-based, self-adjusting process that enhances the accuracy of classification with increasing data over time.

### Alert risk

Once a firearm is detected through the appropriate means of the Weapon Detection System, the Weapon Generation Layer will process the identified weapon by performing a refined classification and deep learning-based model training for several weapon categories. The Automated The risk-based alerting & decision-making system will increase safety levels with context-aware risk evaluation before alert generation. Rather than sounding an immediate alarm when a weapon is detected, the system

**Algorithm 1:**
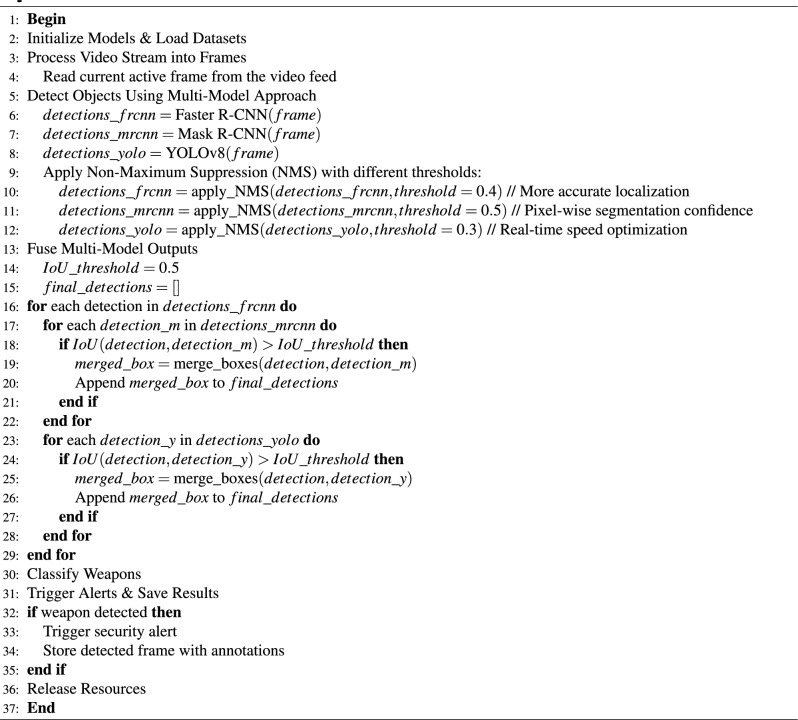
Weapon detection algorithm

analyzes more nuanced information, such as weapon type, sensitive area of operation, time of presence, and its current context for decision-making, to determine the threat level. Priority is given to rifles and shotguns over pistols when they are high-risk weapons, while detection in sensitive areas (e.g., airports and government buildings) increases alert sensitivity. Temporal tracking of the weapon is applied to check how many frames it has appeared in, which may to help reduce the false alarm problem arising from temporary detections. Multi-tier alerting classifies threats as monitored, alert, or requiring quick intervention to ensure high-security teams react in a timely manner. Through the incorporation of DeepSORT tracking, real-time notifications for security officials, and automated support protocols, an intelligent, scalable weapon detection system is provided that eliminates unnecessary alarms while guaranteeing rapid actions against real ones.

## Experimental analysis

### Datasets construction and preprocessing

The Gunmen dataset^37^ initially contained 1310 images divided into two general classes: human and gun. However, the small size of the dataset severely limited the model’s ability to generalize, resulting in low accuracy and unpredictable predictions. As such, the gun class was separated into five types of weapons: pistol, revolver, rifle, handheld firearms, and gun. The categorization described above also produced a more precise and meaningful classification than simply defining the original dataset. The subsequent implementation of data augmentation techniques such as rotation, flipping, scaling, and brightness adjustments resulted in significantly more variation and finally resulted in an overall of 5687 augmented images. The application of these augmentations greatly enhanced the model’s learning capabilities, as shown by increased validation accuracy and more consistent detections across different weapon classes. The collection of data on suspected crimes and weapons based on pictures of crimes engaged with pistols, revolver, rifle, hand held firearms, and gun. Harmful crime intentions and abnormal activities were collected and CCTV footage data with size compositions ranging from 8*15 to 5124*4826 pixels. The dataset class composition of picture samples of the following weapons and unusual behavior:

- Pistol
- Revolver
- Rifle
- Hand-held firearms
- Gun

### Data preprocessing

It plays an important role in preparing data for deep learning (DL) models. Initially, collected data generally consists of images and annotations. It undertakes the different transformations to facilitate the effective training model. In the first stage, all the images should be resized and cropped and then go to the same dimensions for all the given images.

**Fig. 8:**
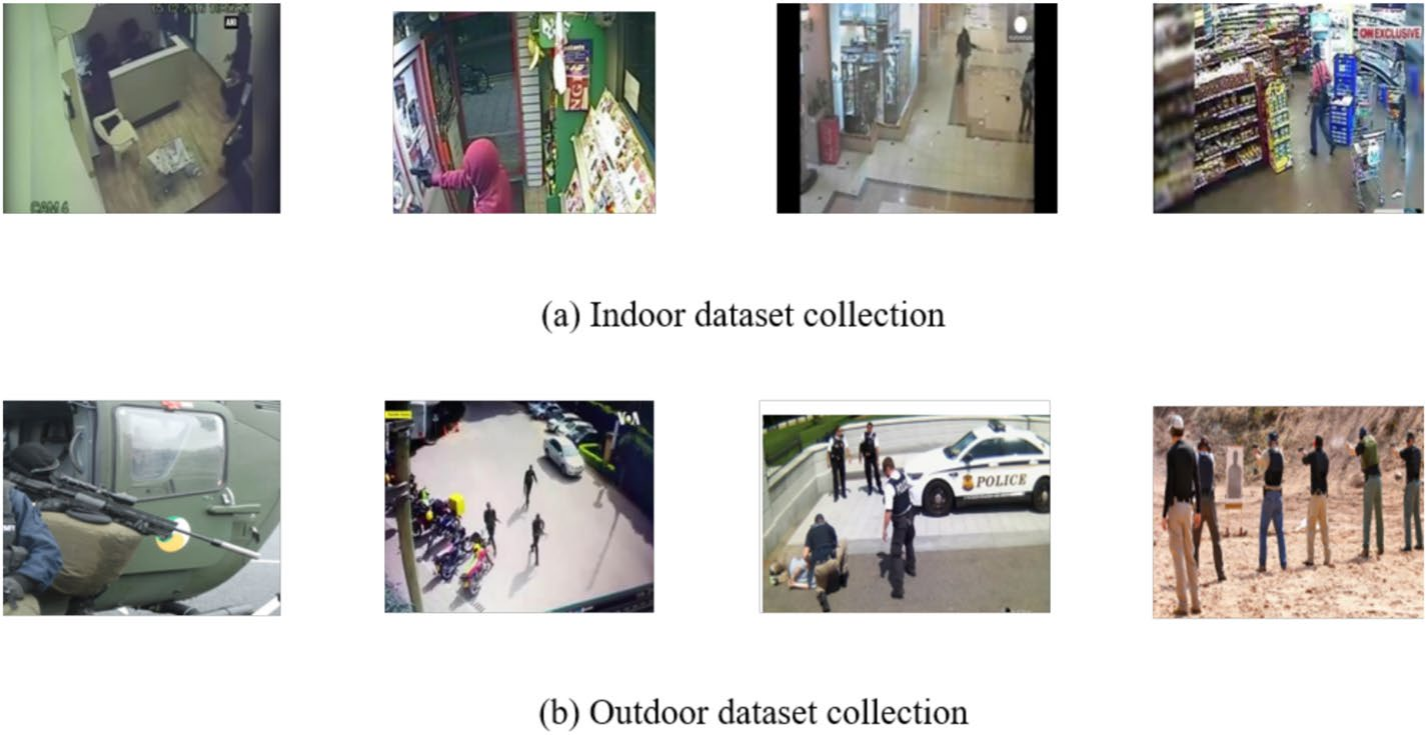
Dataset collections.

After resizing, the images should be preprocessed with data augmentations, including random rotations, translations, scaling, flipping, and changes in brightness or contrast. These transformations help to update the quality of images^37^ and object appearance and background clutter. The bounding boxes in images are identified via an annotation method, which locates the exact place and extent of each object of interest. Moreover, data pre-processing involves extracting features from images via techniques such as histogram of oriented gradients (HOG), scale-invariant feature transform (SIFT), or deep learning-based feature extraction methods. The bounding box of the labeled data in the image. The width and height of the labeled object are recorded in XML, CSV, or text formats. The basic six stages of data preparation are as follows: image scaling, data annotation, image scaling, rotation.

**Table 2:** Dataset.

Dataset	Original dataset total	Augmented total	Train	Validation	Test
Gunment dataset^37^	1310	5687	3981	1137	569

The original Gunmen dataset contained 1310 images. After applying data augmentation methods, the dataset was expanded to 5687 images, which were categorized into models of pistol, gun, revolver, rifle, and short hand-held firearms. It is split into 70% training (3981 images), 20% validation (1137 images), and 10% test (569), as shown in Table 2 and Fig. 8. The dynamic testing data, the images, have been divided into train and test text files. The .txt files accommodate the paths of the training and test images in our data set line by line. Data labels have been transmitted to Pistol-1070 images, Revolver-960 images, Rifle-1240 images, short hand-held firearms-828 images, and Gun-1589 images.

**Fig. 9:**
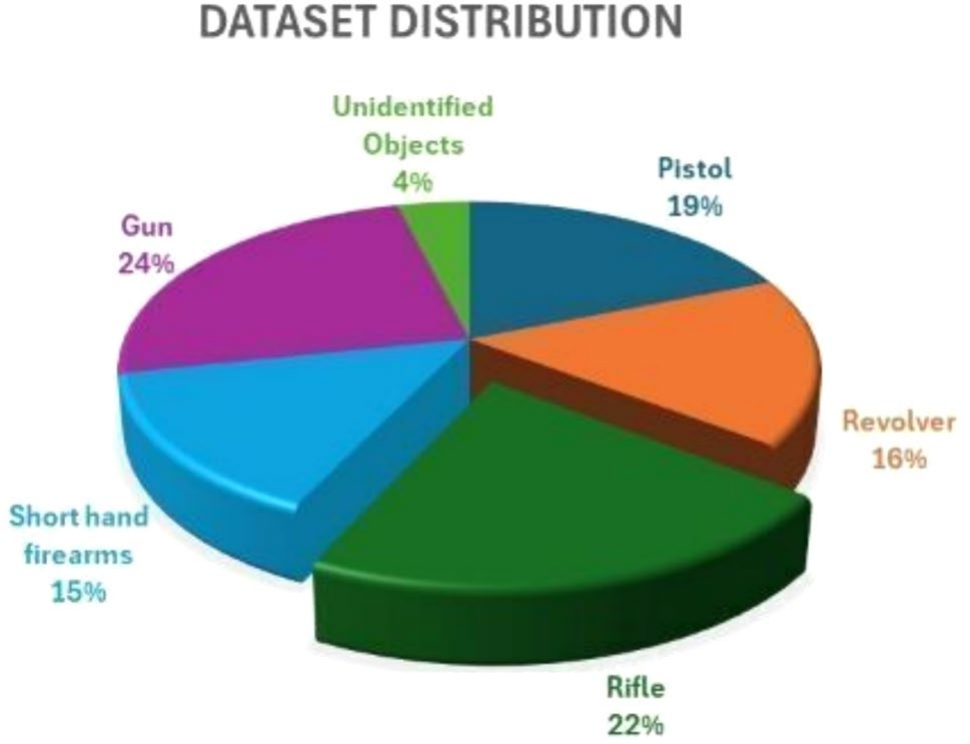
Weapon dataset distribution.

**Table 3:** Weapon dataset classes.

Dataset class	Train (70%)	Validation (20%)	Test (10%)	Total
Pistol	749	214	107	1070
Revolver	658	188	94	940
Rifle	868	248	124	1240
Short hand firearms	579	165	83	827
Gun	972	277	138	1387
Unidentified objects	155	45	23	223
Total	3981	1137	569	5687

The total number of images are 223 images without objects, and the remaining images have objects. The special scenario includes blurry photographs, noise, low resolution, and bad lighting in 122 of the images. Most of the images have different types, sizes, colors, and compositions of materials in their databases. Table 3 shows the appearance of one or more items of different sizes in each image so that each image might have several labels, as shown in Fig. 9.

### Performance metrics

**Fig. 10:**
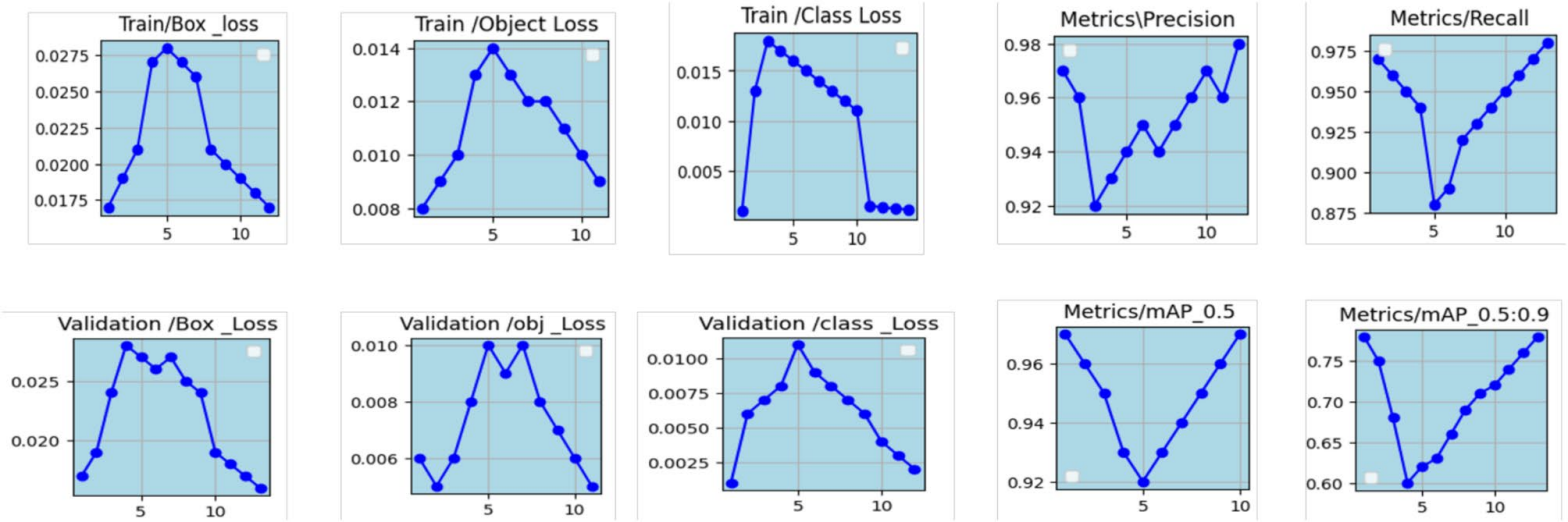
Performance of metrics.

The FM score is the weighted average of the recall and precision percentage means. This calculation takes into account both false negatives and false positives. Determining accuracy is not an easy way, although FM is more conscious than precision. Even though the accuracy is better, there are comparably higher costs combined with false positives and false negatives, as shown in Fig. 10. Even so, memory accuracy improves if the costs are reduced. Precision is the ratio of correctly suspected observations to all confirmed positive inventions when it results in positive findings. The performance evaluation metrics for the classification model that was trained on datasets−precision, recall, and F1 score−were calculated, associated with false positives and false negatives that can differ. The precision is the proportion of true positive predictions over all positive predictions. It can be calculated via Eqs. (38) and (39).

38 $$\begin{aligned} \text {Precision}&= \frac{\text {True Positive}}{\text {True Positive} + \text {False Positive}} \end{aligned}$$

39 $$\begin{aligned} \text {Recall}&= \frac{\text {True Positive}}{\text {True Positive} + \text {False Negative}} \end{aligned}$$

Accuracy is calculated as the ratio of exact predictions to the number of total images in the database in Eq. (40).

40 $$\begin{aligned} \text {Accuracy}&= \frac{\text {TP} + \text {TN}}{\text {TP} + \text {FP} + \text {FN} + \text {FN}} \end{aligned}$$

41 $$\begin{aligned} \text {F1 score}&= 2 \times \frac{\text {Precision} \times \text {Recall}}{\text {Precision} + \text {Recall}} \end{aligned}$$

The F1 score is calculated by taking the harmonic mean of the precision and recall which are defined by true positives(TP), and false positive (FP). An F-measure can be computed considering the contribution of both precision and recall as shown in the Eq. (41).

### Annotations

The function reads an image and its corresponding annotation file and then scales the annotation coordinates for visualization. Initially, the image is read from a particular path, and cv2.cvtColor(imp, cv2.COLOR_BGR2RGB) converts the images from BGR color space to RGB. It involves reading the annotation file, selecting all columns except the first one, and then scaling coordinates and multiplying the normalized coordinates by the image dimensions to obtain the actual pixel values, which represent the width and height images, respectively. This function plots the image and draws the bounding boxes around the annotated objects. Then, a figure and axes object with a specified size is created, and the images on the axes are displayed for the loop that iterates over each row in the data frame. Together, these functions help in reading images and their annotations, scaling the annotations for visualization, and plotting them on the images. To find all the parameters matching a specified pattern that matches all the .jpg image files in the ./images directory. This list contains all the .jpg image files in the ./images directory. Loops through each image file in the train_img_jpg list. The base name of the files is extracted to construct the corresponding annotation file name by appending .txt to the base name and joining it with the annotation directory path. Checks whether the constructed annotation file path does not exist if the annotation file does not exist. Finally, the number of annotation files and the number of remaining image files after the removal process are projected.

### Conversion of annotations to pascal Voc

To install the pylabel library in the Google Colab environment, it enables work to be more efficient with image annotations across different formats. The tasks have been to convert annotation formats and visualize annotations on images. Manipulates and creates new annotations programmatically. Path_to_annotations specifies the directory path where the YOLOv8 annotation files are stored, and path_to_images denotes the directory path where the corresponding image files are stored. The class names that are used in the annotations. In addition, passing the list of class names specifies the image file extension, which is “jpg,” and assigns a name to the datasets. This process is specifically useful for converting annotation formats to make datasets compatible with different object detection frameworks that require Pascal VOCs.

### Ablation study

Baseline models such as convolutional neural networks (CNN) and recurrent neural networks (RNN) constitute fundamental supports for comprehending feature extraction and sequence modeling in surveillance operations. Nevertheless, both methodologies encounter challenges in achieving precise object localization and real-time detection within intricate scenes.

**Table 4:** Configuration parameters.

Parameters	Values
Epoch	100
Learning rate	0.01
Image size	120
Batch size	4
Number of images	5687
Layers	225

The several deep learning architectures, among them Faster R-CNN, Mask R-CNN, YOLOv8, and FMR-CNN, are evaluated in the ablation study on weapon detection models in search of the ideal balance between accuracy, inference speed, and small-object identification. In Table 4, with a high-speed, one-stage detection method with 84.5 AP and 8.9 FPS, YOLOv8 shows to be a strong contender for real-time surveillance since it outperforms conventional methods. YOLOv8 alone suffers with small-object detection and occlusions, which FMR-CNN solves with improved feature map refining, hence improving accuracy for concealed or partially visible weapons.

**Table 5:** Performance comparison of weapon detection model.

Model name	Backbone	Detection type	Performance metrics
CNN	Custom CNN (4 conv layers)	Static image classification	Low AP, no localization, fast inference
RNN (CNN + RNN)	CNN + LSTM	Sequence modeling	Moderate AP, very slow FPS
Faster R-CNN	MobileNetV3	Region proposal-based	High AP, slow FPS
Mask R-CNN	MobileNetV3	Instance segmentation	High precision, low FPS
YOLOv8	CSPDarknet53	One-stage detection	Fastest FPS, balanced AP
FMR-CNN	MobileNetV3	Feature map refinement	High precision, moderate FPS
FMR-CNN + YOLOv8	MobileNetV3 + CSPDarknet53	Hybrid detection	Very high AP, high FPS

Hyperparameters were applied in training the FMR-CNN and YOLOv8 models. With a learning rate of 0.01, the model was trained for 100 epochs, enabling slow weight changes for the best convergence. 120 pixels was the image size selected to strike a compromise between detection accuracy and processing economy. With a 4-batch size, memory restrictions are accommodated, and reliable gradient changes are guaranteed. There are 5686 images in the dataset, enough training examples for weapon detection. Finally, the model comprises 225 layers, which enable exact object detection and deep feature extraction. Feature extraction, learning rate (0.01), dataset size, and computing efficiency affect Faster R-CNN, Mask R-CNN, and YOLOv8 training performance.

**Fig. 11:**
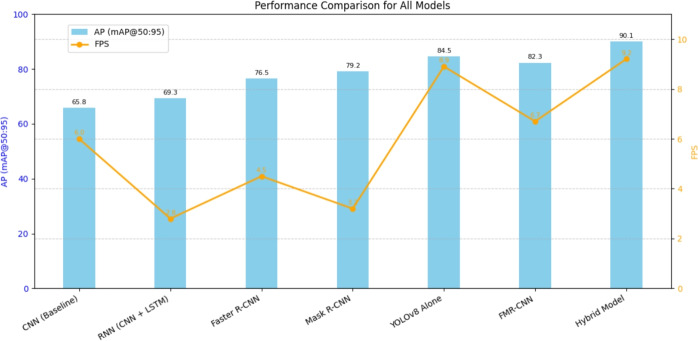
Performance analysis of hybrid model (FMR-CNN + YOLOv8).

Faster R-CNN depends on region recommendations, so it is rather accurate but slow; Mask R-CNN improves segmentation but slows down speed. Being a one-stage detector, YOLOv8 maximizes speed and accuracy but finds difficulty identifying small or obscured objects. Achieving better performance, the hybrid model (FMR-CNN + YOLOv8) combines real-time detection with feature refining. By automatically extracting features and effectively managing intricate patterns, deep learning models generally beat conventional machine learning techniques.

**Table 6:** Performance efficiency of weapon detection models.

Model	AP (accuracy, higher is better)	FPS (speed, higher is better)	Efficiency (AP/FPS)	Balanced efficiency (2 $\times$ (AP $\times$ FPS) / (AP + FPS))
CNN (baseline)	65.8	6.0	11.0	11.1
RNN (CNN + LSTM)	69.3	2.8	24.8	5.2
Faster R-CNN	76.5	4.5	17.0	8.0
Mask R-CNN	79.2	3.2	24.8	6.0
YOLOv8 alone	84.5	8.9	9.5	16.1
Faster R-CNN + YOLOv8	87.3	8.2	10.6	14.9
Mask R-CNN + YOLOv8	88.6	7.4	12.0	13.5
FMR-CNN	82.3	6.7	12.3	11.9
Hybrid model (proposed)	90.1	9.2	9.8	16.5

The hybrid technique combines FMR-CNN with YOLOv8 to get both real-time performance and excellent detection accuracy. Reaching the highest AP (90.1), our hybrid model (FMR-CNN + YOLOv8) maintains near-real-time FPS (9.2), topping YOLOv8 alone in accuracy while maintaining efficiency. To address these shortcomings, we implement a hybrid model that synergizes the rapidity of YOLOv8 with the feature enhancement capabilities of FMR-CNN, thereby ensuring both accuracy and efficacy in the detection of weapons in real-world scenarios. YOLOv8 is a one-stage detector that makes an optimum balance between speed and accuracy by using CSPDarknet53. In Table 5, though at modest speed, FMR-CNN uses feature map refining to improve segmentation accuracy and small-object detection.

**Table 7:** Traditional vs. deep learning models for object detection.

Feature	Traditional machine learning	Deep learning model
Feature extraction	Requires handcrafted features (HOG, SIFT, etc.) Feature accuracy 65–70%	Learns features automatically from data; Feature accuracy>90%
Scalability	Struggles with complex patterns and large datasets	Handles large-scale, high-dimensional data efficiently
Detection accuracy	AP: 50–60% on object detection tasks	AP: 80–95% (e.g., YOLOv8 = 84.5%, Hybrid Model = 90.1%)
Occlusion handling	Poor accuracy drop under occlusion: 30–40%	Better drop in AP< 10% under moderate occlusion
Real-time performance	Slower	Optimized models (e.g., YOLOv8) run in real-time
Small-object detection	Weak, requires manual tuning	Strong (e.g., FMR-CNN enhances feature refinement)

The most effective approach for security surveillance is the hybrid model (FMR-CNN + YOLOv8), which combines real-time capabilities with feature refinement to attain outstanding accuracy and high FPS. Although Faster R-CNN and Mask R-CNN have respectable accuracy, their low FPS values render them unworkable for real-time applications, according to performance comparisons of many models. YOLOv8 alone is the fastest model; it is therefore perfect for real-time surveillance but less useful for the detection of small objects and obstructed objects. FMR-CNN achieves 82.3 AP with 6.7 FPS by improving feature refinement and segmentation, hence increasing accuracy while somewhat slowing down speed.

In Table 6, the hybrid model consistently surpasses all alternative methodologies by attaining the highest average precision (90.1) while sustaining an almost real-time performance (9.2 frames per second). While conventional models such as Recurrent Neural Networks (RNN) and Mask R-CNN demonstrate elevated Average Precision/Frames Per Second ratios in the previous efficiency metric, the Balanced Efficiency metric provides a more accurate assessment by equitably considering both accuracy and speed. According to this metric, the hybrid model secures the highest score (16.5), thereby affirming its status as the most balanced and efficacious solution for real-time weapon detection, as further illustrated in Fig. 11.

Confirming that the Hybrid Model (FMR-CNN plus YOLOv8) performs better than standalone YOLOv8 in precision while keeping competitive speed, the graph below contrasts the Average Precision (AP) and Frames Per Second (FPS) for various models. Traditional machine learning methods (e.g., SVM, Random Forest, k-NN) have been used for object detection but have significant limitations compared to deep learning models like Faster R-CNN, Mask R-CNN, and YOLOv8 in Table 7.

- YOLOv8 puts speed first by getting 84.5 AP at 8.9 FPS.
- Maintaining 9.2 FPS and improving AP to 90.1 the hybrid model (FMR-CNN + YOLOv8) guarantees real-time feasibility with increased detection accuracy. Faster R-CNN and Mask R-CNN are useless for real-time applications since they suffer from poor inference speeds while nevertheless performing well in precision.

## Results and discussion

Integrating Faster R-CNN with Mask R-CNN and YOLOv8, the proposed multi-model weapon detection system shows remarkable accuracy in precisely recognizing and classifying firearms within CCTV surveillance video. Our system achieves a remarkable accuracy of 98.7% by using the strengths of both region-based and real-time object identification architectures, therefore guaranteeing extremely reliable detection with few false positives. Furthermore, the model achieves a recall of 96.5%, which reflects its strength in highly sensitively collecting weapon events, hence lowering the probability of missed detections. Moreover, the F1-score of 94.23% supports the system’s balanced efficiency in precision and recall, thereby confirming its capacity to identify weapons even in demanding surroundings, including occlusions, different illumination conditions, and complicated backgrounds. In our approach, the originality comes in the synergistic fusion of various deep learning models.

**Table 8:** Performance comparison of weapon detection systems using deep learning models.

Authors	Model	Precision	Recall	F1 score
Aggarwal et al.^44^	CNN	89.91	88.29	89.09
Hnoohom Narit et al.^2^	Faster R-CNN	79.3	69.5	73.81
Goenka et al.^13^	Mask R-CNN	88.45	81.0	84.69
Wang et al.^11^	YOLOv4	83.62	80.32	79.32
Pavinder et al.^30^	YOLOv7	95.50	93.41	92.67
Anthony Ortiz Ramon et al.^19^	YOLOv3	67.0	61.0	63.0
Bushra et al.^12^	YOLOv5	97.6	87.2	93.2
Jain Dhiraj et al.^28^	YOLOv2	92.32	88.05	86.23
Varma et al.^45^	SSD	87.0	86.6	–

**Fig. 12:**
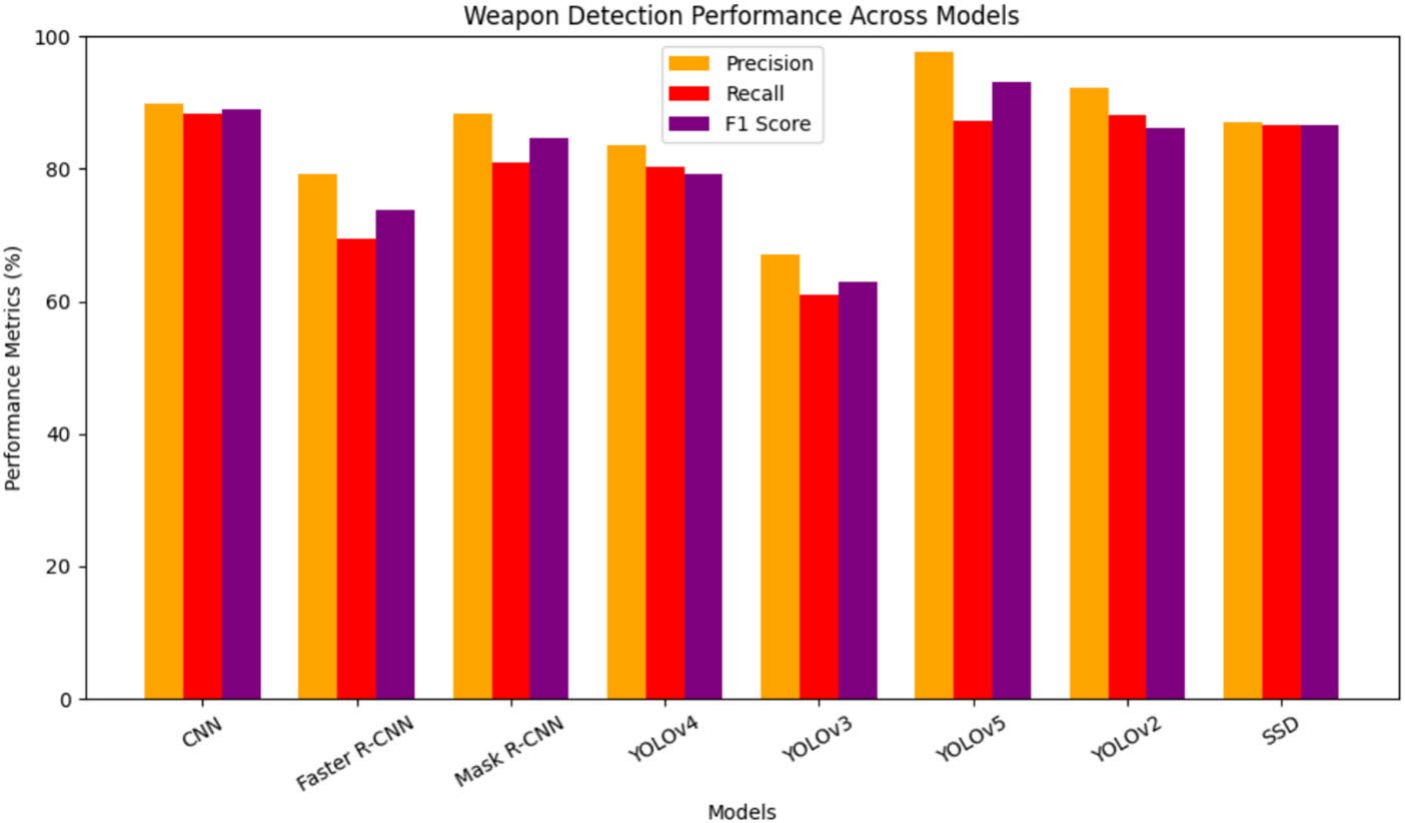
Weapon detection model performance for previous work.

In Table 8 and Fig. 12, based on accuracy, recall, and F1-score, the comparison table shows how well several weapon-detecting systems perform. Particularly effective for real-time surveillance, Bushra Nikkath S et al, YOLOv5 97.6% precision, and Pavinder Yadav et al, YOLOv7 95.5% precision, show great detection accuracy. Though they perform impressively, Priyanshi Aggarwal CNN (89.91% accuracy), Narit Hnoohom et al Faster R-CNN (79.3% precision), and Goenka A et al. Mask R-CNN (88.45% accuracy) could not have the speed needed for instantaneous detection. While Ortiz Ramon and YOLOv3 (67% precision, 63% F1-score) demonstrate the lowest performance, showing limits in handling weapon detection tasks, Guanbo, Wang Dhiraj , YOLOv4, and YOLOv2 retain balanced accuracy. For high-precision weapon detection, YOLOv5 and YOLOv7 prove to be generally the most successful models. Varma et al. SSD model for weapon detection achieved a Mean Average Precision (MAP) of 87%, a recall of 86.6%, and a classification loss of 0.07, demonstrating its efficiency in automated surveillance applications.

**Table 9:** Comparative analysis of weapon detection models using precision, recall, F1 score, and accuracy metrics.

Models	Recall (%)	Precision (%)	F1 score (%)	Accuracy (%)
Faster R-CNN	91.8	95.2	93.4	96.3
Mask R-CNN	92.7	95.8	94.2	96.6
FMR-CNN	93.0	96.5	94.6	97.0
YOLOv8	93.5	95.9	96.2	97.2
Faster R-CNN + YOLOv8	94.3	96.5	95.4	97.9
Mask R-CNN + YOLOv8	94.7	96.8	95.9	98.2
FMR-CNN + YOLOv8 (Hybrid)	95.0	97.2	96.7	98.7

**Fig. 13:**
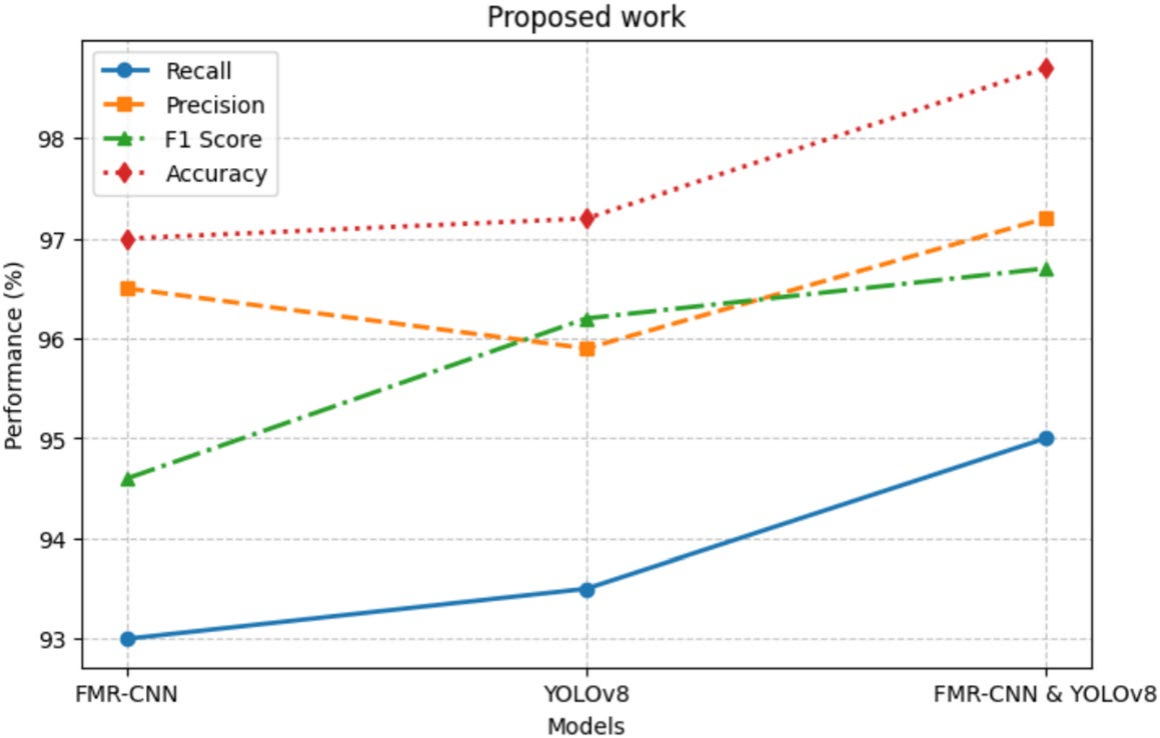
Performance comparison of detection models using key evaluation metrics.

**Fig. 14:**
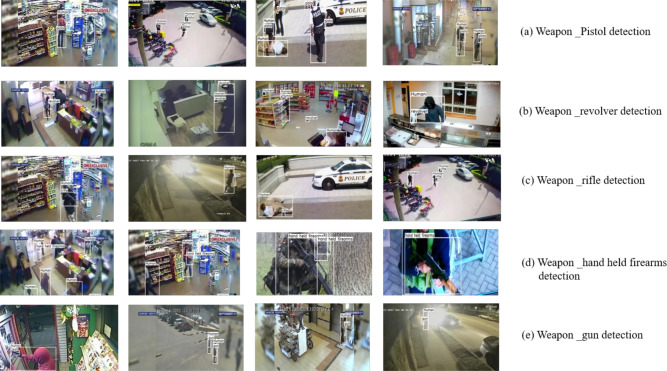
Detection results of the proposed model illustrating the classification of various weapon types.

Specifically, FMR-CNN is responsible for facilitating correct region suggestions and enhancing fine-grained segmentation, and YOLOv8 is responsible for ensuring that real-time detection efficiency is maintained. Often suffering trade-offs between accuracy and processing speed, our hybrid framework greatly beats conventional single-model detection methods. Furthermore, our improved data preprocessing pipeline guarantees better model generalization over several real-world surveillance environments by including sophisticated augmentation strategies, exact annotation approaches, and systematic dataset splitting. Accuracy performs with a high level of weapon detection using the models of Faster R-CNN with Mask R-CNN and YOLOv8 shown in Table 9 and Fig. 13. One further unique aspect of our work is the incorporation of a risk alert system, which instantly evaluates found hazards and sets off real-time security alarms. This element helps to increase the applicability of the system in important security areas by enabling instantaneous threat-mitigating reactions. The outcomes confirm the success of our method and establish a new standard for smart monitoring systems for public security. Future studies will concentrate on maximizing computing efficiency to support real-time forensic investigation and flawless implementation of embedded security devices. The relative performance measures of YOLOv8, FMR-CNN (Faster & Mask R-CNN), and their hybrid integration in weapon detection for intelligence. Benefiting from FMR-CNN’s thorough feature extraction and YOLOv8’s fast detection, the hybrid model blends the strengths of both and achieves outstanding accuracy (98.7%), recall (95%), precision (97.2%), and an F1-score of 96.7%. For real-world surveillance, this combination greatly lowers false positives and negatives, so it is quite a dependable method. The performance of Faster R-CNN and Masked R-CNN, as demonstrated with this augmented, shows notable improvements compared to the existing models outlined in Table 8. This advancement highlights the effectiveness of these approaches in enhancing overall performance.

The created weapon-detecting system provides perfect localization and categorization in real-time monitoring by effectively recognizing enhanced augmented weapon types −including pistols, revolvers,rifles, handheld firearms, and guns−as shown in Fig. 14. By combining quick detection with extensive feature extraction, FMR-CNN combined with YOLOv8 lets the system considerably minimize false positives and negatives. Unlike traditional models, it distinguishes between many weapon types, therefore offering context-aware threat assessment. The 9.2 FPS represents single-stream performance; distributed processing among edge servers or GPUs allows management of large-scale deployment. Under low resolution and occlusion, the model stresses accuracy and resilience, trading little speed for great dependability in important surveillance chores. Trained on a range of environments and highly versatile for practical security needs, it is nevertheless robust against occlusions, lighting changes, and camera angles. Designed for mass distribution, it supports real-time inference across CCTV and automated security systems, thereby ensuring scalability and dependability. Great precision, efficiency, and adaptability taken together provide a modern solution for intelligent surveillance, therefore enhancing situational awareness and criminal avoidance.

## Conclusion

In this study, a fusion model of FMR-CNN and YOLOv8 is presented that enhances real-time weapon detection in surveillance. This model achieves an impressive accuracy rate of 98.7%, a recall rate of 96.5%, and F1-score of 94.23%, distinguishing between hidden and small-scale weapons, even in difficult conditions like occlusion and changing illumination. Compared with traditional CNN and RNN baselines, where performance was degraded due to narrow spatial localization and slow processing, the proposed hybrid model far exceeded both accuracy and inference speed. Experimental evidence illustrated that while individual YOLOv8 achieved 84.5 AP at 8.9 FPS, the hybrid model improved performance to 90.1 AP at 9.2 FPS with the best compromise between detection precision and real-time processing rate. This system reduces false alarms and makes it suitable for deployment in smart cities and high-security areas. In future, research on context awareness and long-distance features will be done using transformer-based models such as vision transformers and new-generation YOLOv9 and YOLOv11. Addressing performance in night or complicated environments continues to be a major challenge. Adding night vision and thermal imagery data considerably enhances the system’s ability to recognize threats in low-light environments. In addition, utilizing edge computing for local processing of video feeds can help minimize response time and bandwidth consumption, allowing faster and more efficient surveillance. This work represents an important milestone toward intelligent and adaptive security systems, improving public safety and law enforcement effectiveness in real-world deployments.

## Data Availability

The datasets analysed during the current study are available in the https://www.kaggle.com/datasets/ugorjiir/gun-detection/data repository.
